# Therapeutic targeting of chromatin alterations in leukemia and solid tumors

**DOI:** 10.1002/ijc.35389

**Published:** 2025-04-03

**Authors:** Florian Perner, Tobias Berg, Daniel Sasca, Sophie‐Luise Mersiowsky, Jayant Y. Gadrey, Johanna Thomas, Michael W. M. Kühn, Michael Lübbert

**Affiliations:** ^1^ Hematology, Hemostasis, Oncology and Stem Cell Transplantation Hannover Medical School (MHH) Hannover Germany; ^2^ DGHO, Deutsche Gesellschaft für Hämatologie und Medizinische Onkologie e.V. Working Group ‘Clinical and Translational Epigenetics’ Berlin Germany; ^3^ Centre for Discovery in Cancer Research, Faculty of Health Sciences McMaster University Hamilton Ontario Canada; ^4^ Department of Hematology and Medical Oncology University Medical Center, Johannes Gutenberg‐University Mainz Germany; ^5^ Department of Hematology, Oncology and Stem Cell Transplantation University Medical Center Freiburg, Faculty of Medicine Freiburg Germany; ^6^ Department of Medicine Tufts Medical Center Boston Massachusetts USA

**Keywords:** epigenetic therapy, histone methylation, menin inhibition

## Abstract

Alterations in chromatin conformation and post‐translational modification of histones have become increasingly recognized as critical drivers of cancer development, progression, and therapy resistance. Recent advances in drug development have led to the establishment of several highly selective small molecule inhibitors, several of which are currently under investigation in clinical trials. These compounds allow the precise interrogation of specific chromatin features and functions, transforming the field of epigenetic cancer therapy from rather unselective approaches, like histone deacetylase inhibition or demethylating agents, toward the use of true targeted therapeutics. Recently, several compounds altering histone methylation have been investigated in preclinical and clinical trials. We summarize the current state of development for inhibitors against Menin‐KMT2A, DOT1L, KDM1A, and Polycomb complexes, and the arginine methyltransferase PRMT5 for the treatment of myeloid malignancies, lymphoma, and different solid tumors. Inhibitors of IDH1/2 act at the interface of epigenetic and metabolic vulnerabilities and have been FDA‐approved for the treatment of IDH‐mutant glioma, cholangiocarcinoma, and myeloid malignancies. Several clinical trials investigating effective combination therapy regimens are ongoing and summarized herein. Drugs targeting components of SWI/SNF chromatin remodeling complexes, such as inhibitors of SMARCA4, have entered the clinic. Members of SWI/SNF complexes are among the most frequently mutated genes across all cancer entities, and novel selective inhibitors for the first time allow pharmacologic interrogation of aberrant chromatin remodeling. Overall, the therapeutic interrogation of chromatin‐related processes plays an increasing role in cancer therapy. This chapter reviews recent developments and future directions in the field of epigenetic cancer therapy.

## Graphical abstract

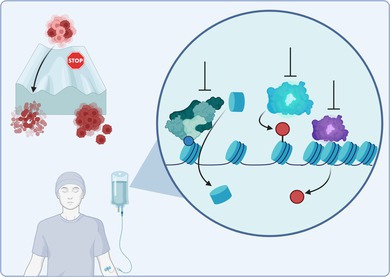

## 1 HISTONE METHYLTRANSFERASES AND EPIGENETIC GENE REGULATION

Histone methylation is crucial in the precise temporal and spatial coordination of DNA‐templated processes.^1^ The histone methylation landscape is maintained by a diverse group of enzymes catalyzing the addition of methyl groups most commonly to lysine or arginine residues at histone tails. These enzymes catalyze methylation at key lysine residues on histones, including canonical sites such as lysine 4 (H3K4), lysine 9 (H3K9), lysine 27 (H3K27), lysine 36 (H3K36), and lysine 79 (H3K79) on histone H3, and lysine 20 (H4K20) on histone H4, thereby regulating a wide array of chromatin functions.^1^, ^2^ Histone methyltransferase enzymes exhibit explicit selectivity for specific histone lysine residues and the degree of methylation (e.g., mono‐, di‐, or trimethylation) that they confer.^3^, ^4^ This specificity makes them attractive targets for the development of selective drugs. The catalytic activity is conferred by a conserved domain, while accessory proteins govern the selectivity and activity of the complex, fine‐tuning the enzyme's function.^3^

In the human proteome, two primary domains are associated with methyltransferase activity: the “Su(var)3–9, Enhancer‐of‐zeste and Trithorax” (SET) domain and the seven‐beta‐strand (7βS) domain. The SET domain, named after the Drosophila proteins Su(var)3–9, enhancer of zeste, and trithorax, is present in 55 human proteins and selectively allows lysine methyltransferase (KMT) activity.^1^, ^2^, ^5^ Approximately half of these proteins are active KMTs, methylating histone and non‐histone substrates. The 7βS family is larger and more diverse, with about 150 members in humans. These proteins target many substrates, including lysine, arginine, other amino acids, N‐terminal α‐amines, and nucleic acids. A total of 24 enzymes, 23 SET proteins and one 7βS protein, generate the canonical histone lysine methylation marks.^1^, ^2^, ^3^, ^5^

Dysregulation of KMT activity caused by mutations, chromosomal translocations, or altered gene expression is commonly linked to cancer and developmental disorders.^1^ Recently, significant progress has been made in developing promising chemical compounds that inhibit specific KMTs in various cancer types, with several of these drugs now being tested in clinical trials.^6^, ^7^, ^8^

### 1.1 Targeting the Polycomb‐repressive complex 2

The regulation of the balance between activating and repressive chromatin methylation marks is essential for defining cell identity during development and differentiation and plays a critical role in cancer. One of the first settings in which the importance of this balance has been exemplified is the control of bivalent developmental genes in Drosophila melanogaster by the competition between Trithorax‐group activators and Polycomb repressors.^9^ The mammalian homologs of these protein families are KMT2A‐ and (polycomb‐repressive complex 2) PRC2‐complexes, which are critical in controlling homeobox (HOX) transcription factor gene expression during development and are frequently altered in various cancers.^9^, ^10^, ^11^, ^12^ The polycomb repressive complex 2 (PRC2) has methyltransferase abilities at lysine 27 at histone H3, leading to the formation of H3K27me3. The H3K27me3 histone mark is generally associated with transcriptional repression.^9^ The core complex required for the catalytic function of PRC2 comprises EZH1/2, zinc finger protein SUZ12, and the WD40 protein EED.^13^, ^14^ Activating mutations or high‐level EZH2 expression are common in cancer, making EZH2 a promising therapeutic target.^15^, ^16^

Preclinical data suggest that sensitivity to EZH2 inhibition depends on molecular settings that create PRC2 dependencies. For example, activating EZH2 mutations occur in 27% of patients with follicular lymphoma and 22% of diffuse large B‐cell lymphoma (DLBCL),^17^ drive the disease, and represent a specific target for pharmacologic inhibition.^15^, ^18^, ^19^ Furthermore, high EZH2 expression is also observed across various cancer types, offering additional therapeutic avenues.^15^, ^16^, ^20^, ^21^ Additionally, mutations in other chromatin‐modifying complexes can create synthetic lethality with EZH2 inhibition. In cancers driven by mutations in the SWI/SNF chromatin remodelling complex, such as rhabdoid sarcoma and small cell carcinoma of the ovary, EZH2 inhibition exploits a specific dependency on PRC2 function,^22^, ^23^, ^24^ vide infra.

The efficacy of EZH2 inhibition in various preclinical cancer models, and initially conducted with 3‐Deazaneplanocin A (DZNep)^25^ provided the rationale for evaluating Tazemetostat, a selective EZH2 inhibitor, in patients with relapsed/refractory lymphomas or various solid tumors.^26^ Overall, Tazemetostat had a favorable safety profile, with asthenia being the most common treatment‐related side effect (33% of patients). Specific histological cancer subsets were noted to have more favorable responses to Tazemetostat. B‐cell lymphomas showed a high overall response rate (ORR) of 38%, including some complete remissions (CR).^26^ In epithelioid sarcoma, the ORR was lower, but some patients had stable disease and remained on treatment for more than 20 months.

Based on the encouraging activity of Tazemetostat against B‐cell lymphomas in phase I of NCT01897571, investigators aimed to investigate further whether specific lymphoma entities would exhibit enhanced efficacy and whether EZH2 mutational status would influence treatment sensitivity. Thus, a phase II single‐arm trial (NCT01897571) evaluated Tazemetostat monotherapy in patients with relapsed‐refractory follicular lymphoma.^27^ Ninety‐nine patients were enrolled and were divided into two cohorts: 45 patients were recruited to the EZH2 mutant (mut) cohort, while 54 were recruited to the EZH2 wildtype (WT) cohort. Overall, the drug was well tolerated, with thrombocytopenia (3%) and neutropenia (3%) being the most common treatment‐related grade 3 adverse events. Clinically meaningful responses were observed with Tazemetostat monotherapy. The ORR of 69% and 35% was seen in the EZH2^mut^ and the EZH2^wt^ cohort, respectively. Based on this study, the US FDA granted Tazemetostat an accelerated approval in June 2020 for the treatment of relapsed‐refractory EZH2^mut^ follicular lymphoma. Reassuringly, another phase II study evaluated Tazemetostat in a Japanese lymphoma cohort and reported an ORR of 76% in EZH2^mut^ follicular lymphoma patients,^28^ hence similar to that seen in NCT01897571.^29^

Although EZH2^mut^ patients appeared to have enhanced sensitivity to Tazemetostat in this trial, a subsequent study re‐evaluated the data to estimate the outcome after variations in the baseline characteristics were minimized. A propensity score was created for each participant with EZH2^mut^ or EZH2^wt^ status based on the likelihood of being selected based on their baseline characteristics. Participants were subsequently matched using a 1:1 nearest neighbor approach. Intriguingly, this analysis suggested that the outcomes between the EZH2^mut^ and EZH2^wt^ may have been similar had the cohorts been more closely matched, indicating that the genotype‐specific therapeutic benefit in the EZH2^mut^ patients may have been overestimated.^30^

As noted above, mutations in the SWI/SNF chromatin remodeling complex (e.g., in the SMARCB1 gene) create context‐specific vulnerability to EZH2 inhibition, and durable responses were seen in the Tazemetostat phase I trials in epithelioid sarcomas harboring a loss of SMARCB1 (albeit in very limited sample size of 2 out of 3 patients),^26^ a basket trial was designed to assess Tazemetostat in solid tumor patients harboring loss of INI1/SMARCB1. Of 62 patients with INI1/SMARCB1 deleted/mutated epithelioid cancers, the ORR was 15% at data cut‐off, and an additional 26% exhibited disease control at 32 weeks of treatment. Based on these phase II data, Tazemetostat also received accelerated approval by the FDA in January 2020 for the treatment of locally advanced or unresectable metastatic epithelioid sarcoma in the US.^29^ A confirmatory phase Ib/III trial evaluating the efficacy of Tazemetostat combined with doxorubicin was designed and is currently recruiting patients (NCT04204941); see also Table 4.

The efficacy of Tazemetostat was also evaluated in BRCA‐1 associated protein 1 (BAP1) inactivated cancers as preclinical data suggest that BAP1 inactivation leads to enhanced EZH2 expression associated with pronounced sensitivity to EZH2 inibition.^31^ These findings provided the rationale to assess Tazemetostat as a monotherapy in malignant pleural mesothelioma that is known to exhibit BAP1 inactivation in up to two‐thirds of cases. In a phase I/II single‐arm study (NCT02860286) assessing Tazemetostat in 61 patients, disease control (primarily disease stabilization) was achieved in 54%.^32^

While the previous studies evaluated Tazemetostat monotherapy, two studies have been published to date assessing Tazemetostat‐based combination therapies. A phase Ib open‐label study (NCT02220842) evaluated the empirical combination of Tazemetostat with the checkpoint inhibitor Atezolizumab in diffuse large B‐cell lymphoma (DLBCL). This study enrolled 48 patients and noted only a modest response: the objective response rate (CR + PR) was noted in only 16% of patients, while stable disease was noted in an additional 16% of patients.^33^ In a second phase Ib trial (NCT02889523) Tazemetostat was empirically combined with the R‐CHOP standard of care regimen for patients with newly diagnosed DLBCL. Reported response rates were encouraging, with a metabolic complete remission (mCR) of 75%, warranting further evaluation of this combination.^34^

While Tazemetostat is the clinically most developed drug of its class, other EZH2 inhibitors listed in Table 1 are under early clinical investigation.^35^, ^36^, ^37^ These include published results from clinical trials evaluating GSK2816126, SHR2554, and DS‐3201b (now known as Valemetostat). A phase I trial (NCT02082977) evaluating GSK2816126 established the MTD of 2400 mg, but the pharmacological profile was found to limit effective exposure. Consistent with this, GSK2816126 induced only modest responses, with a partial remission (PR) in a single patient and stable disease as the best response in 14 of 41 patients.^35^ SHR2554, another EZH2 inhibitor, was assessed in a phase I trial (NCT03603951) in patients with relapsed or refractory mature lymphoid neoplasms. In the exploratory efficacy cohort, an ORR of 43% was noted, warranting further evaluation in future studies.^38^ Lastly, the EZH2 inhibitor Valemetostat was evaluated in a phase II trial (NCT04102150) after two phase I trials established the recommended phase 2 dose (RP2D).^39^ This study was designed to evaluate the efficacy of Valemetostat in patients with relapsed or refractory adult T‐cell leukemia/lymphoma. An encouraging ORR of 48% was observed in this difficult‐to‐treat patient cohort, warranting further investigation of Valemetostat in patients with adult T‐cell leukemia/lymphoma.^40^ Less promising are the results from one phase I/II combination study combining Valemetostat with irinotecan in patients with recurrent small cell lung cancer (SCLC); unfortunately, this combination was not tolerated, and future combination studies shall be designed to avoid cumulative toxicity.^41^

**TABLE 1:** Clinical trials of PRC2‐complex inhibitors.

NCT Number, IIT	Interventions	Phases	Enrollment	Disease	Setting
EPZ6438 (Tazemetostat)					
NCT02875548	*EPZ6438*	I & II	100	Solid tumors (including synovial sarcoma, epitheliod sarcoma, mesothelioma, renal medullary carcinoma) DLBCL, NHL	N/A
NCT04179864 IIT	*EPZ6438*, abiraterone/prednisone, enzalutamide	I & II	102	Prostate cancer	Metastatic and/or castration resistant
NCT04846478	*EPZ6438*, talazoparib	Ia & II	35	Prostate cancer	Metastatic and/or castration resistant
NCT04204941	*EPZ6438*, Doxorubicin	III	164	Phase Ib: soft tissue sarcoma; Phase III: epitheliod sarcoma	Locally advanced and unresectable or metastatic
PF‐06821497 (Mevrometostat)					
NCT03460977	*PF‐06821497*, enzalutamide	I	343	Prostate cancer (currently recruiting arms)	Metastatic, unresectable and/or castration resistant (currently recruiting arms)
NCT06551324	*PF‐06821497*, docetaxel, enzalutamide	III	600	Prostate adenocarcinoma	Metastatic, unresectable and/or castration resistant
NCT06629779	*PF‐06821497*, enzalutamide	III	900	Prostate adenocarcinoma	Metastatic, unresectable and/or castration resistant
SHR2554					
NCT03603951	*SHR2554*	I	272	Mature lymhphoid neoplasms, Peripheral T cell lymphomas, DLBCL, Multiple Myeloma, Waldenstrom macroglobulinemia	Resistant, no standard therapy available
NCT06368167	*SHR2554*	II	105	Follicular lymphoma	Relapsed/refractory
NCT06173999	*SHR2554*, CHOP, CHOEP	I & II	100	Peripheral T cell lymphomas	N/A
NCT04407741 IIT	*SHR2554*, SHR 1701	I & II	100	NSCLC (adenocarcinoma and SCC), Pancreatic adenocarcinoma, Cholangiocarcinoma, Gastrointestinal adenocarcinoma, TNBC, B‐cell lymphoma	Relapsed/refractory
NCT05896046 IIT	*SHR2554*, SHR 1701	I & II	100	Hodgkin Lymphoma	Relapsed/refractory
NCT06568094	*SHR2554*, HRS‐5041, HRS‐1167, Docetaxel, Prednisone, Abiraterone	I & II	100	Prostate Adenocarcinoma	Advanced
NCT05559008 IIT	*SHR2554*, Apatinib, Camrelizumab, Tucidinostat, Linperlisib, Dasatinib, Azacitidine	I & II	116	Peripheral T cell lymphomas	Relapsed/refractory
NCT04355858 IIT	*SHR2554*, (SHR7390, Famitinib, SHR3162, Pyrotinib, Capecitabine, SHR1210, Everolimus, Nab paclitaxel, SHR3680, SHR6390, SHR1701, SERD, AI, VEGFi)	II	319	HR+/HER2‐ invasive (luminal) breast cancer	Locally advanced, recurrent metastatic
NCT06519526 IIT	*SHR2554*, SHR‐0302 Ivarmacitinib	II	25	Peripheral T cell lymphomas	Relapsed/refractory
NCT06122389	*SHR2554*, Tucidinostat	III	130	T cell lymphoma	Relapsed/refractory
DS‐3201b (Valemetostat)					
NCT02732275	*DS‐3201b*	I	100	Non‐Hodgkin's Lymphoma	Relapsed/refractory
NCT04102150	*DS‐3201b*	II	25	Adult T cell Leukemia/Lymphoma	Relapsed/refractory
NCT04842877 IIT	*DS‐3201b*	II	141	B‐cell non‐HL, Aggressive B cell lymphomas, Follicular lymphomas, MCL, MZL, Indolent lymphomas, HL	Relapsed/refractory
NCT04703192	*DS‐3201b*	II	155	Peripheral T cell lymphomas, Adult T cell lymphomas	Relapsed/refractory
NCT06244485	*DS‐3201b*, T‐DXd, Dato‐DXd	I	210	Solid tumors: HER2‐positive gastric cancer, Non‐squamous NSCLC, HER2 low breast cancer	Unresectable/metastatic/relapsed/refractory
NCT06294548 IIT	*DS‐3201b*, atezolizumab, bevacizumab	I & II	45	HCC	Locally advanced, metastatic, or unresectable
MAK683					
NCT02900651	*MAK683*	I & II	139	NHL Solid tumors	NHL: relapsed/refractory; Solid tumors: advanced or recurrent/metastatic
ORIC‐944					
NCT05413421	*ORIC‐944*, abiraterone, apalutamide, darolutamide, enzalutamide	I	250	Prostate cancer	Metastatic

*Note*: Overview of ongoing clinical trials involving inhibitors of PRC2‐complexes, specifically the enzymatic subunit EZH2.

Abbreviations: DLBCL, diffuse large B cell lymphoma; HCC, hepatocellular carcinoma; HER2, human epidermal growth factor receptor 2; HL, Hodgkin lymphoma; HR, hormone receptor; MCL: mantle cell lymphoma, MZL, marginal zone lymphoma; NHL, non‐Hodgkin lymphoma; NSCLC, non‐small cell lung cancer; PRC2, polycombrelated complex 2; SCC, squamous cell carcinoma; TNBC, triple‐negative breast cancer.

While the inhibitors reviewed thus far target the catalytic domain of EZH2, an alternative strategy of disrupting the methyltransferase function of PRC2 may be realized via disruption of the core catalytic complex of PRC2, which, as described above, comprises EZH1/2, SUV12, and EED. This alternative strategy has been realized through the development of the EED inhibitor MAK‐683, an allosteric inhibitor of EED; the binding of MAK‐683 causes a conformational change that disrupts the interaction between EED and EZH2, consequently disrupting the methyltransferase function of EZH2.^42^ The phase I/II clinical trial evaluating MAK‐683 in adult patients with advanced malignancies (NCT02900651) is currently recruiting patients, but results have not yet been presented. Ongoing clinical trials assessing drugs that disrupt PRC2 complex functions are provided in Table 1.

### 1.2 The Menin–KMT2A complex as a clinical drug target

The mammalian Trithorax homolog KMT2A (formerly known as MLL or MLL1) is a histone methyltransferase and contributes to promoter activation by catalyzing H3K4me3, thereby supporting transcriptional activation.^43^, ^44^, ^45^ KMT2A is affected by gain‐of‐function mutations in various cancers and by chromosomal translocations leading to the formation of oncogenic fusion proteins in the so‐called *KMT2A*‐rearranged leukemias (KMT2A‐r), a distinct leukemia subtype. Aberrant activation of KMT2A‐target genes, part of a conserved stem‐cell program, is driven by KMT2A‐fusion oncogenes or activated by wild‐type KMT2A complexes as demonstrated specifically in *NPM1* ^mut^ leukemias.^8^, ^46^, ^47^, ^48^, ^49^, ^50^ *KMT2A*‐rearrangements occur in about 10% of acute leukemia patients and are associated with a particularly poor prognosis, while NPM1^mut^ are present in about 30% of patients with acute myeloid leukemia and are linked to relatively favorable outcomes after chemotherapy.^51^ While efforts to therapeutically target the KMT2A histone methyltransferase directly were not successful, it is now possible to indirectly target KMT2A by inhibiting its protein interaction with the adaptor protein Menin. Some general aspects of therapeutic Menin inhibition will be discussed here, whereas a separate article in this special issue of the journal will provide a more detailed overview.

Menin, an adaptor protein, binds to the N‐terminal portion of KMT2A and modulates its chromatin interactions. Disrupting the Menin–KMT2A interaction through genetic inactivation or pharmacological inhibition compromises the integrity of oncogenic KMT2A–chromatin complexes.^48^, ^52^, ^53^, ^54^, ^55^, ^56^, ^57^, ^58^

In preclinical model systems, different Menin inhibitors have demonstrated impressive efficacy in *KMT2A*‐r, *NPM1* ^mut^, and *NUP98*‐rearranged leukemia^50^, ^52^, ^54^, ^55^, ^59^, ^60^, ^61^, ^62^, ^63^, ^64^ leading to a rapid translation into early‐phase clinical trials. The Menin inhibitor field is currently developing rapidly, and we will focus this review section on Revumenib, the molecule that is currently most advanced on the route to FDA approval. Other molecules and clinical studies are discussed in a separate review article in this special issue.

The AUGMENT‐101 trial investigated the Menin inhibitor Revumenib (SNDX‐5613) in patients with relapsed or refractory *KMT2A*‐r and *NPM1* ^mut^ leukemia.^65^ At the inception of the study, patients with relapsed/refractory acute leukemia, regardless of cytogenetic and mutational profile, could enroll; however, enrollment was subsequently restricted to *KMT2A*‐r and *NPM1* ^mut^ leukemia given the strong evidence that those biological subsets critically depend on the Menin–KMT2A interaction. At the time of reporting results, 68 patients were enrolled. Overall, Revumenib was well tolerated, with reversible QTc prolongation being the most prevalent treatment‐emergent adverse event. At the time of enrollment, patients had received a median of four prior therapies, and 46% had relapsed after allogeneic stem cell transplantation. The importance of using preclinical evidence to inform enrollment criteria is reflected in the response rates: among evaluable patients, the overall response rate was 53%, and the complete remission (CR) or complete remission with partial hematologic recovery (CRh) rate was 30%. The median duration of response was 9.1 months for patients with CR or CRh.^65^ The published phase II data from the same trial show similarly promising results: The efficacy‐evaluable cohort (*n* = 57) showed a CR + CRh rate of 22.8, with an ORR of 63.2%, and 68.2% of patients in CR achieved undetectable residual disease. Given these early and encouraging response rates, Revumenib received priority review status from the FDA for approval in the US and was approved for MLLr leukemias on Nov. 15, 2024.

### 1.3 Small molecule inhibitors of DOT1L and H3K79 methylation

DOT1L stands out as the sole histone methyltransferase capable of catalyzing the methylation of histone H3 at lysine 79 (H3K79me1, ‐me2, and ‐me3).^66^, ^67^, ^68^ This modification is distributed throughout the gene body and is closely associated with actively transcribed genes. In adult mammals, the function of DOT1L is generally non‐essential for the homeostasis of most cell types, with the notable exception of hematopoietic stem cells.^69^ KMT2A‐fusion oncogenes in acute leukemias either recruit DOT1L directly through their fusion partners, such as AF9, AF10, AF17, or ENL, or indirectly to their target genes, thereby fostering an active chromatin environment that counteracts transcriptional repression.^70^, ^71^, ^72^, ^73^, ^74^, ^75^, ^76^ Recent evidence also suggests that DOT1L may play a role in stabilizing the KMT2A–fusion chromatin complex.^63^

Consequently, pharmacological inhibition of DOT1L has emerged as a strategic approach to target the transcriptional programs driven by KMT2A fusion and KMT2A proteins harboring a partial tandem duplication (PTD) mutation in KMT2A.^77^ Given that DOT1L's methyltransferase activity is not required in normal tissues, its pharmacological inhibition represents a highly selective therapeutic strategy in the context of *KMT2A*‐rearranged and other KMT2A‐dependent leukemias. The first selective inhibitor of DOT1L, EPZ004777, was reported in 2011 and was shown to reduce H3K79me levels and cell proliferation markedly.^78^ Further structural optimization led to the development of the second‐generation DOT1L inhibitor, Pinometostat (formerly known as EPZ5676), which demonstrated enhanced potency and selectivity. Notably, in a rat xenograft model, Pinometostat resulted in complete tumor regression after 3 weeks of administration, with no significant toxicity reported.^79^

Despite the pharmacological challenges associated with Pinometostat, this agent was evaluated as a monotherapy in a Phase 1 clinical trial targeting relapsed or refractory KMT2A‐r acute myeloid leukemia (AML) (NCT01684150).^80^ The trial enrolled a total of 51 patients with KMT2A‐r leukemia, organized into six dose‐escalation and two expansion cohorts. Consistent with findings from preclinical studies, Pinometostat was administered via continuous infusion over a 28‐day period. Steady‐state plasma concentrations were attained within 4–8 h of initiating the infusion, but levels diminished rapidly upon cessation of treatment. Overall, the regimen was well tolerated, and a maximum tolerated dose was not established, partly due to limitations related to the drug's solubility and infusion rate. Only three of the 51 patients who received the treatment completed the trial. The predominant reason for discontinuation was disease progression, affecting 31 patients (60%). Although two participants achieved complete remission while on the medication, they ultimately experienced a relapse shortly after finishing the last cycle of Pinometostat.^80^

Since Pinometostat was well tolerated at biologically efficacious doses (able to reduce H3K79me levels) and lacked significant single‐agent activity, investigators designed two clinical trials to evaluate the efficacy of Pinometostat‐based combination therapy regimens. The clinical trial NCT03701295 assessed Pinometostat and Azacytidine, while the other (NCT03724084) evaluated Pinometostat and 7 + 3 induction chemotherapy (cytarabine and daunorubicin). Both trials have been completed and are currently being analyzed.

In summary, DOT1L inhibition holds promise as a valuable therapeutic target for myeloid malignancies and, potentially, other cancers. Recent advances in structure‐guided optimization have produced a series of DOT1L inhibitors with improved pharmacokinetic profiles, including reduced plasma concentration requirements and enhanced oral bioavailability. These novel compounds now allow preclinical researchers to assess combination therapies using DOT1L inhibitors with other epigenetic agents and define doses for reaching effective in vivo inhibition in animal models.^81^, ^82^ These studies will address the medical need for the preclinical development of biology‐informed combination therapy regimens.

### 1.4 Targeting PRMT5—a methyltransferase with highly complex, cell‐type‐specific regulation and function

PRMT5 is a member of the protein arginine methyltransferase (PRMT) family of enzymes whose canonical function is to serve as “writers” of the post‐translation modification of arginine methylation. While three distinct types of methylation have been characterized thus far, PRMT5 writes the catalyzed ω‐NG—monomethyl‐arginine (MMA) and the ω‐NG, NG—symmetric dimethylarginine (sDMA) mark by transferring a methyl group from S‐Adenosylhomocysteine (SAM) to the protein target.^83^ Like other chromatin‐modifying enzymes, PRMT5 functions as a part of a protein complex: PRMT5 exists as a part of a hetero‐octameric complex, comprising four subunits of PRMT5 and four subunits of WDR77/MEP50.^84^

PRMT5 has been thus far characterized to methylate H2AR3, H3R2, H3R8, H3R2, and H4R3; the symmetrical methylation of these residues has been associated with the regulation of gene expression.^85^, ^86^, ^87^ The function of PRMT5 is further fine‐tuned by way of modulation of PRMT5 expression levels by microRNA,^87^, ^88^ modulation of PRMT5 protein stability by ubiquitination,^28^ post‐translational modifications of PRMT5 itself (methylation and phosphorylation),^89^, ^90^, ^91^ and protein–protein interactions.^83^ This intricately complex regulation influences the PRMT5 protein level, sub‐cellular localization, protein targets (both histone and non‐histone) for arginine methylation, and consequently, the cellular function of PRMT5 in a context‐specific manner. As a direct consequence, PRMT5 has been shown to regulate diverse cellular processes including the regulation of gene expression, alternative splicing, mRNA translation, DNA damage response, cell signaling, and cell metabolism. The mechanistic roles of PRMT5 in these diverse cellular processes have been reviewed in detail elsewhere.^83^

PRMT5 has been demonstrated to play a particularly critical role in leukemia and myeloproliferative neoplasms. In acute myeloid leukemia (AML), PRMT5 silences miR‐29b expression by H4R3me2, which leads to upregulation of transcription factor “Specificity protein 1” (SP1) and activation of the “Fms‐like tyrosine kinase 3” (FLT3).^92^ Furthermore, PRMT5‐mediated methylation of the splicing factor SRSF1 is a critical event to safeguard alternative splicing of critical oncogenic transcripts in AML.^93^ In Acute Promyelocytic Leukemia (APL), PRMT5 contributes to resistance against arsenic trioxide treatment via another non‐histone related mechanism. In APL, PRMT5 methylates RNF4, thereby stabilizing the oncogenic fusion protein PML‐RARA.^94^ The two‐faced role of PRMT5 in blood cancer pathogenesis is exemplified by its role in myeloproliferative Neoplasms (MPN). In JAK2‐mutant classic MPN, constitutively active JAK2 binds to PRMT5 and phosphorylates it. This phosphorylation disrupts the association of PRMT5 with its co‐activator MEP50, thereby impairing its methyltransferase activity. In this setting, reduced PRMT5 activity was shown to play a role in the development of classic MPN.^95^ In contrast, PRMT5 is overexpressed in Chronic Myeloid Leukemia (CML) downstream of oncogenic BCR‐ABL signalling and its inactivation causes a substantial proliferative disadvantage in CML stem cells.^96^

The diversity of targets of PRMT5‐driven methylation has important implications for drug development. As mentioned previously, PRMT5 is responsible for methylating the arginine residues on both histone and non‐histone proteins. While developing therapies to inhibit PRMT5‐driven methylation, the cellular consequences of target engagement must be evaluated with the caveat that they may not necessarily be related to the reduction of histone arginine methylation. The complexity of discerning the etiology of the response to PRMT5 inhibition is reflected in the secondary outcomes of PRMT5 inhibitor clinical trials. The currently active PRMT5 inhibitor clinical trials, as listed in Table 2, do not have a secondary outcome that evaluates a biomarker of response that would imply that the observed efficacy is due to abrogating or inhibiting cellular processes that are critically dependent upon histone arginine methylation. To our knowledge, thus far no clinical trial study has published data linking the efficacy of PRMT5 inhibition to a reduction in histone arginine methylation levels.

**TABLE 2:** Active clinical trials of PRMT5 inhibitors.

NCT number	Interventions	Phases	Enrollment	Disease	Setting
NCT05528055	SCR‐6920	I	122	Metastatic solid tumors, NHL	Solid tumors: locally advanced or metastatic; NHL: relapsed/refractory
NCT05732831	TNG‐462	I &II	159	MTAP‐deleted solid tumors	Locally advanced, metastatic, and/or unresectable
NCT05275478	TNG‐908	I & II	192	MTAP‐deleted solid tumors and GBM	Solid tumor: Locally advanced, metastatic, and/or unresectable, GBM: relapsed/refractory
NCT06130553	AZD3470	I & II	210	MTAP‐deleted solid tumors	Recurrent or metastatic
NCT06137144	AZD3470	I & II	110	Hematologic malignancies	Relapsed/refractory
NCT05245500	MRTX1719	I & II	580	MTAP‐deleted solid tumors	Unresectable or metastatic
NCT06333951	AMG‐193, carboplatin, paclitaxel, pembrolizumab pemetrexed, sotorasib	I	500	MTAP‐deleted NSCLC (including squamous, non‐squamous, PD‐L1+, and KRAS G12C‐mutant)	Locally advanced or metastatic
NCT06360354	AMG‐193, Gemcitabine, Nab‐Paclitaxel, modified FOLFIRINOX	I	180	MTAP‐deleted gastrointestinal, biliary tract, pancreatic cancers	Locally advanced (and unresectable) and/or metastatic
NCT03573310	JNJ‐64619178	I	114	Solid tumors, B‐cell non‐Hodgkin Lymphoma, lower risk MDS	B‐cell NHL: relapsed/refractory, Solid tumors: advanced
NCT06589596	BGB‐58067	I	92	MTAP‐deleted or MTAP‐deficient solid tumors	Advanced, metastatic, or unresectable
NCT05094336	AMG‐193, Docetaxel	I & II	649	CDKN2A‐deleted and/or MTAP‐deleted or MTAP‐deficient solid tumors	Locally advanced and unresectable or metastatic

*Note*: Active clinical trials of PRMT5 inhibitors.

Abbreviations: CDKN2A, cyclin‐dependent kinase inhibitor 2A; MDS, myelodysplasic syndrome; MTAP, methylthioadenosine phosphorylase; NHL, non‐Hodgkin lymphoma; NSCLC, non‐small cell lung cancer; PD‐L1, programmed cell death ligand 1.

### 1.5 Conclusions and future directions

Histone methyltransferases (HMTs) play a pivotal role in the regulation of epigenetic processes through the precise modulation of histone methylation. These enzymes target specific lysine and arginine residues on histone tails, catalyzing mono‐, di‐, or tri‐methylation to influence chromatin structure and gene expression. Canonical sites such as H3K4, H3K9, H3K27, H3K36, H3K79, and H4K20 are critical nodes of this regulation, with their methylation states linked to diverse chromatin functions. The selectivity of HMTs for specific residues and methylation states makes them attractive candidates for therapeutic targeting. Emerging pharmacotherapeutic strategies are focusing on targeting HMTs, and several inhibitors are currently under clinical investigation. Ongoing clinical trials are exploring the potential of small‐molecule inhibitors for HMTs such as EZH2, which is implicated in various cancers, including lymphomas and sarcomas. Promising results from early‐phase trials have demonstrated the efficacy and tolerability of EZH2 inhibitors in specific patient populations, but overall, the therapeutic efficacy remains modest.

Menin inhibitors are a novel class of targeted therapies under active clinical investigation for their potential in treating acute leukemias, particularly KMT2A‐rearranged and NPM1‐mutant leukemias. These compounds work by disrupting the interaction between Menin and KMT2A, a HMT catalyzing H3K4‐methylation, which drives leukemogenesis. Early‐phase clinical trials have shown promising response rates, demonstrating significant anti‐leukemic activity with manageable safety profiles. Of note, the first Menin inhibitor, Revumenib, was approved for the treatment of relapsed and refractory leukemia with KMT2A‐rearrangements or NPM1 mutations in November 2024.

Preclinical and mechanistic studies further suggest that combined targeting of specific HMTs could greatly enhance therapeutic efficacy. For example, a dual inhibitor of EZH2 and Euchromatic histone‐lysine N‐methyltransferase 2 (EHMT2) showed a significant increase in potency in breast and ovarian cancer, as well as in acute myeloid leukemia, while retaining a favorable safety profile and preserving, even enhancing, antitumor immunity in vivo.^97^, ^98^, ^99^ Similarly, only combined inhibition of Menin and DOT1L phenocopied experimental degradation of the KMT2A‐fusion protein and globally silenced oncogenic KMT2A‐driven gene expression in leukemia^63^ providing a strong rationale for pursuing combined targeting of oncogenic HMT complexes in future clinical trials.

## 2 THERAPEUTIC TARGETING OF KDM1A MEDIATED HISTONE DEMETHYLATION

### 2.1 Mechanistic studies and preclinical results

Lysine‐specific demethylase 1 (LSD1 or KDM1A) is a histone demethylase that is highly evolutionarily conserved and serves as an important hub for chromatin remodeling complexes. In a pioneering study, it was discovered as the first enzyme to mediate histone demethylation in 2004.^100^ Even before this role was identified, it had been found as part of several co‐repressor complexes, for example, the REST corepressor complex (CoREST) and the Nucleosome remodelling and deacetylase (NuRD) complex.^101^, ^102^, ^103^, ^104^, ^105^, ^106^ It acts as a flavin‐adenine dinucleotide (FAD)‐dependent monoaminoxidase and can catalyze the demethylation of histone H3 lysine 4 (H3K4)^100^ and histone H3 lysine 9 (H3K9).^107^ While H3K4 di‐methylation is an activating histone mark and is mostly found, for example, on active enhancers, H3K9 di‐methylation is found in the context of heterochromatin formation and gene repression. Depending on the context, LSD1 can thereby act to activate or repress transcription.

The activating role of LSD1 was first found in the context of androgen‐receptor‐dependent transcription.^107^ The repressor function of LSD1 is mediated by its ability to interact with the CoREST complex, which includes histone deacetylases. In this context, the presence of LSD1 thereby leads to a reduction in H3K4 di‐methylation and H3K27 acetylation, which has been demonstrated using CAS9‐fusions.^108^, ^109^ The targeting of this repressive activity is mediated not by direct DNA interaction but often by the physical interaction of LSD1 with SNAG domain transcription factors (such as GFI1 or GFI1B)^110^ (Reviewed in^111^).

Germline knockout mice of LSD1 exhibit embryonic lethality before embryonic day E7.5 and exhibit the earliest severe developmental defects.^112^, ^113^ LSD1 has been found to be an important regulator in numerous cellular differentiation processes as diverse as hematopoietic differentiation,^114^, ^115^ adipocyte differentiation and function,^116^, ^117^ effector T cell differentiation^118^ and neural differentiation.^119^ The conditional knock‐out of LSD1 in hematopoietic stem cells leads to severe pancytopenia mediated by disrupting terminal differentiation. This is accompanied by a failure to downregulate genes associated with stem cell function (such as Meis1).^114^ Interestingly, transient inhibition of LSD1 has been found to facilitate an expansion of hematopoietic stem cells *in vivo* ^115^ and in vitro.^120^ Disruption of CoREST function, including LSD1, through degradation mediated by the CLR3‐KBTBD4 complex was important for the activity of the potent stem cell expansion agent UM171.^121^

While LSD1 has not been found mutated, it has been found overexpressed in many different types of cancers such as prostate,^122^ breast,^123^ small cell lung cancer (SCLC),^124^ medulloblastoma^125^ and hematological cancers, for example, in the context of acute myeloid leukemia (AML)^126^ (Reviewed in^127^). Based on this and its crucial role in differentiation processes, numerous studies investigate LSD1 inhibition in different types of cancer. In particular, in AML and SCLC, there is an increasing understanding of the specific biological activity of LSD1 inhibition, and important clinical trials of LSD1 inhibition have been conducted in these entities.

In two groundbreaking preclinical AML studies, LSD1 inhibition has been found to lead to cellular differentiation and a reduction in leukemic stem cells.^128^, ^129^ While usually not active in this non‐APL AML, LSD1 inhibition has also sensitized AML cells to the effect of All‐Trans Retinoic Acid (ATRA), suggesting the use of LSD1 inhibition as a combination treatment. The activity of LSD1 inhibition in AML is specifically related to the displacement of LSD1 from its interaction partner GFI1.^130^ Importantly, not all inhibitors of LSD1 mediate this effect, but it was found mostly with irreversible inhibitors such as tranylcypromine and its derivatives^131^ that bind covalently to the FAD coenzyme, which binds in proximity to the SNAG interacting domain. Interestingly, the modulation of the enzymatic activity of LSD1 appears dispensable for the effect of LSD1 inhibition in AML.^130^, ^131^

LSD1 inhibition can modulate myeloid transcription factor networks (reviewed in^132^). Crucial in this context are important myeloid transcription factors CEBPA and PU.1, which have both been shown to be relevant for the activity of LSD1 inhibition.^133^ Another transcription factor modulated by LSD1 inhibition in AML is the monocyte transcription factor IRF8.^131^, ^134^ In a murine model system, the cell‐of‐origin further played a role in the response of AML cells to LSD1 inhibition. These results have been connected to the overexpression of the early hematopoietic transcription factor EVI1, as well as the activity of p53. High EVI1 expression and loss of p53 negatively influenced the response of AML cells to LSD1 inhibition. The effect of high EVI1 expression can be overcome by combining LSD1 inhibition with Venetoclax, a first rationale for this combination.^135^

Pioneering work using preclinical models of myeloproliferative diseases led to an expansion of the role of LSD1 inhibition in these diseases, where it was shown to prolong survival in murine disease models by reducing the clone size.^136^ LSD1 inhibition has further been demonstrated to be a relevant target in Small‐Cell Lung Cancer (SCLC). Screening a broad panel of cell lines derived from different cancers, Mohammad et al. found a particular activity in AML and SCLC cells.^137^ Response to LSD1 inhibition in SCLC was associated with specific methylation signatures.^138^ This effect has been attributed to disrupting the interaction of LSD1 with the SNAG domain proteins INSM and GFI1B^139^ and to the activation of Notch1 by LSD1 inhibition.^140^ Newer preclinical data further suggest a relevant activity of LSD1 inhibition in enhancing the activity of checkpoint inhibitors in SCLC.^141^, ^142^

Another cancer where LSD1 is directly involved in the pathogenesis of the disease is medulloblastoma. A subset of medulloblastoma is driven by the activation of GFI1. Inhibition of LSD1 is active in this subset of medulloblastoma.^1^ LSD1 inhibition has been implicated in neuroblastoma due to its high expression and direct interactions with the transcription factor MYT1.^143^, ^144^. In addition to its roles in cancer, LSD1 inhibition has also been postulated in neurodegenerative disorders (in particular, Alzheimer's), where LSD1 has been implicated in neuroinflammation, autophagy, the balance of neurotransmitters, and the progression of the tauopathy phenotype (reviewed in^145^). One important consideration in the context of targeting these conditions is the greater relevance of avoiding on‐target hematopoietic side effects of LSD1 inhibition caused by the interference with the GFI1 and GFI1B interaction of LSD1.

### 2.2 Clinical development of KDM1A inhibitors

#### 2.2.1 Acute myeloid leukemia

The first clinical trials of LSD1 inhibition in AML were initiated with the use of tranylcypromine (TCP), which has long been in clinical use as a MAO inhibitor for the treatment of depression; see Table 3. TCP inhibits LSD1 with an IC50 value of 20.7 μM. Importantly, it inhibits MAO A and MAO B with lower IC50 values of 2.3 and 0.95 μM, respectively.^146^ In total, three clinical trials were conducted, all of which combined the use of tranylcypromine with ATRA. Patients included in these trials were patients with relapsed and refractory AML and MDS. One trial (NCT02261779, conducted in Halle and Heidelberg by Carsten Müller‐Tidow) included a rapid ramp‐up of tranylcypromine to 60 mg daily over the course of 6 days, after which ATRA was initiated at a dose of 45 mg/m^2^ in two divided doses. The use of hydroxyurea for count control was permissible. The trial included 18 patients and showed an overall response rate of 20% (including 2 CRi and 1 PR). Median survival was 3.3 months, and overall survival at 1 year was 22%. Interestingly, as a sign of biological activity, one case of differentiation syndrome was observed.^147^ Another trial (NCT02273102, conducted at the University of Miami by Justin Watts) employed a 3 + 3 dose escalation design with increasing doses of TCP (10 mg BID, 20 mg BID, 30 mg BID). This trial observed a maximum tolerated dose (MTD) of 20 mg BID (also with a rapid dose escalation). ATRA was given at a dose of 45 mg/m^2^ in two divided doses. The trial included 17 patients and observed an overall response rate of 23.5% (including 1 MLFS, 2 SD with HI, 2 SD). Median survival was 5.0 months.^148^ The third trial (NCT02717884, TRANSATRA, multicentre trial conducted under the lead of Michael Lübbert) used a rolling‐six design and four dose levels of TCP (20, 40, 60, and 80 mg p.o. on days 1–28) with a slow ramp‐up dosing (in 10‐mg steps q 4 days) in combination with ATRA (45 mg/m^2^ p.o. on days 10–28) and low‐dose cytarabine (40 mg s.c. on days 1–10). It has been reported in abstract form and included 25 patients^149^ and demonstrated a similar response rate to the two other trials. While some transcription data in the Miami trial suggested an enhanced ATRA effect, the reported pharmacokinetic data from the Heidelberg trial cast doubt on the possibility that an effective level of LSD1 inhibition is achievable with TCP at clinically relevant concentrations, reinforcing the need for the development of more potent and more specific LSD1 inhibitors.

**TABLE 3:** Clinical trials of LSD1 inhibitors in AML, MPN, and SCLC.

Indication	Trial	Compound	Patient Number	Outcome	Reference
AML (relapsed/refractory)	NCT02261779	Tranylcypromine (TCP: Non‐selective, irreversible monoamine oxidase inhibitor) + ATRA	18	2 CRi, 1 PR	Wass et al., 2020^147^
AML and MDS (relapsed/refractory)	NCT02273102	Tranylcypromine + ATRA	17	1 MLFS, 2 SD with HI, 2 SD	Tayari et al., 2021^148^
AML and MDS (relapsed/refractory)	NCT02717884 (TRANSATRA)	Tranylcypromine + ATRA	25		In preparation^149^
AML (relapsed/refractory)	EudraCT 2013‐002447‐29	Iadademstat (selective, irreversible KDM1A inhibitor)	25	1 CRi	Salamero et al., 2020^150^
AML (newly diagnosed)	EudraCT 2018‐000482‐36	Iadademstat + azacitidine	36	14 (52%) of 27 patients CR/CRi	Salamero et al., 2024^151^
AML (relapsed/refractory)	NCT06357182	Iadademstat + azacitidine + Venetoclax	Ongoing		
AML (relapsed/refractory)	NCT05546580	Iadademstat + azacitidine + Venetoclax	Ongoing		
AML and MDS (relapsed/refractory)	NCT02842827	Bomedemstat (selective, irreversible KDM1A inhibitor) + ATRA	45	1 PR of 39 patients with AML	Disclosed on ClinicalTrials.gov ^276^
AML and MDS (relapsed/refractory)	NCT05597306	Bomedemstat + Venetoclax	Ongoing		
Myelofibrosis	NCT05569538	Bomedemstat + Ruxolitinib	Ongoing		
ET (resistant to or intolerant of at least one standard treatment)	NCT04254978	Bomedemstat	73	76.6% response at endpoint (95% response overall)	Goethert et al., ASH 2023^155^ Disclosed on ClinicalTrials.gov
Myelofibrosis	NCT03136185	Bomedemstat	90	66% Spleen Volume Reduction	Gill et al., ASH 2022^154^ Disclosed on ClinicalTrials.gov
ET	NCT06456346	Bomedemstat vs. Hydroxyurea	Ongoing		
ET (inadequate response to hydroxyurea)	NCT06079879	Bomedemstat vs. BAT	Ongoing		
SCLC	NCT02034123	GSK2879552 (selective, irreversible KDM1A inhibitor)	29	CNS SAEs	Bauer et al., 2019^156^
SCLC 2 L	EudraCT 2018‐000469‐35	Iadademstat in combination with platinum/etoposide	14	40% PR 20% long‐lasting SD	Mendivil et al., 2020^157^
SCLC and extrapulmonary high grade NET (relapsed)	NCT05420636	Iadademstat + Paclitaxel	Ongoing		
SCLC ED	NCT06287775	Iadademstat + Atezolizumab or Durvalumab	Ongoing		
SCLC ED	NCT05191797	Bomedemstat + Atezolizumab	3 Terminated		
Advanced malignancies	NCT02712905	INCB059872 (selective, irreversible KDM1A inhibitor) + ATRA, azacitidine, nivolumab	116	0% AML 1/13 AML with ATRA 1/7 AML with Aza 20% SCLC	Disclosed on ClinicalTrials.gov

*Note*: Summary of clinical trials of LSD1 inhibitors in AML, MPN, and SCLC.

Abbreviations: AML, acute myeloid leukaemia; ATRA, all‐trans‐retinoic acid; Aza, Azacytidine; BAT, best available therapy; CR, complete remission; CRi, complete remission with incomplete haematological recovery; ED, extensive disease; ET, essential thrombocythemia; LSD1, lysine‐specific histone demethylase 1A; MDS, myelodysplasic syndromes; MLFS, morphologic leukemia‐free state; PR, partial remission; SD, stable disease; SD HI, stable disease with hematologic improvement; SAE, serious adverse event; SCLC, small‐cell lung cancer.

Iadademstat (ORY‐1001) is a highly selective and potent covalent inhibitor of LSD1. It inhibits LSD1 at an IC50 of <20 nM. It was tested in a phase I single‐agent trial on 25 patients with relapsed and refractory AML. Dose escalation was performed in a traditional 3 + 3 design. The recommended dose of this compound was 140 μg/m^2^/d as a single agent given for 5 days every 28 days. The trial observed 1 CRi and 2 reductions in blast count (formally not counting as a response). The trial also demonstrated upregulation of expected LSD1 targets, such as CD86, consistent with a strong on‐target activity.^150^ These encouraging data led to an open‐label, phase 2a dose‐finding study in combination with azacytidine in newly diagnosed patients with AML (ALICE).^151^ The study included a 3 + 3 dose‐finding phase. Patients received iadademstat orally for 5 days on and 2 days off weekly, with azacitidine 75 mg/m^2^ subcutaneously for 7 of 28 days. The recommended phase 2 dose of iadademstat in this setting was 90 μg/m^2^ per day. The trial included 36 patients. 82% of the efficacy data set achieved an objective response (22/27 patients). 14 (52%) of 27 patients had complete remission or complete remission with incomplete hematological recovery. This composite complete remission rate is higher than with single‐agent azacitidine but lower than with the current standard combination of azacitidine and venetoclax reported in the Viale‐A trial (28.3% and 66.4%, respectively).^152^

Bomedemstat (IMG7289) is a highly selective and potent inhibitor of LSD1. It was tested in patients with AML and MDS with moderate single‐agent activity comparable to Iadademstat (unpublished). A combination trial with Venetoclax is ongoing (VenBom, NCT05597306).

With combination data with Azacitidine and preclinical data suggestive of a high degree of synergism between LSD1 inhibition and Venetoclax,^135^, ^153^ studying LSD1 inhibition in combination with Azacitidine and Venetoclax is the next natural step in advancing this treatment principle for patients with AML. Additional trials are ongoing in combination with the FLT3 inhibitor Gilteritinib.

#### 2.2.2 Myeloproliferative neoplasias

Bomedemstat is currently mostly developed in myeloproliferative neoplasias (MPN), where it was shown to control the disease symptoms and directly affect the size of the malignant clone. These results have so far mostly been presented in abstract form. A phase 2 study has been completed in advanced myelofibrosis (*N* = 89). Of patients evaluable for spleen volume reduction at 24 weeks (*N* = 50), 66% had a spleen volume reduction from baseline. Of transfusion‐independent patients (*N* = 41), 90% had stable or improved hemoglobin. Of those requiring transfusions, 14% became independent.^154^ Making use of the strong observed reduction in platelet count upon LSD1 inhibition, Bomedemstat has also been tested for Essential Thrombocythemia (ET) with a high response rate (95%) and importantly relevant reductions in clone size.^155^

#### 2.2.3 Small‐cell lung cancer

Based on the promising preclinical data in Small‐cell lung cancer (SCLC) discussed above, a phase 1 dose‐escalation trial has been conducted with GSK2879552 in relapsed/refractory SCLC (NCT02034123). The trial included 29 patients, of whom 22 completed it.^156^ The results of the trial were disappointing, particularly due to severe toxicities that led to the discontinuation of the compound's development. The main concerns were severe thrombocytopenia (41%) and, even more concerning, severe encephalopathy.

Iadademstat has also been tested in SCLC in the CLEPSIDRA trial in second‐line Extensive Disease (ED)‐SCLC patients (EudraCT n° 2018‐000469‐35) who were candidates for re‐challenge chemotherapy and had confirmed positive expression of Iadademstat responsive biomarkers. This trial has been completed and presented in abstract form, with objective responses observed.^157^ Trials in combination with Paclitaxel (NCT05420636) and immune checkpoint inhibitors (NCT06287775) are ongoing and planned. Bomedemstat was studied in combination with Maintenance Immunotherapy (Atezolizumab) for Treatment of Newly Diagnosed ED‐SCLC (NCT05191797). The results of this study were made available. INCB059872 and CC‐90011 were additional LSD1 inhibitors undergoing early‐phase evaluation for efficacy in cancer. These programs have been terminated and are currently not active.

#### 2.2.4 Neurodegenerative disorders

The only active clinical development program for an LSD1 inhibitor outside the cancer field is currently the program for Vafidemstat (ORY‐2001). A first‐in‐human study has demonstrated good tolerability,^158^ and the compound is currently under development in a spectrum of neuropsychiatric disorders.

In summary, LSD1 inhibition is promising in AML and SCLC, and additional preclinical data make it interesting also in other cancers. Based on its activity, it will be necessary to develop LSD1 inhibitors as part of combinations and integrate them with current standard treatment. Identifying biomarkers that could predict a response to the combination is of great interest.

### 2.3 Conclusions and future directions

LSD1 inhibition shows significant promise in AML and SCLC, with emerging preclinical data suggesting its potential in other cancers. While initial trials have demonstrated relatively low response rates with single‐agent LSD1 inhibitors, their use in combination strategies is still in its early stages. Developing LSD1 inhibitors as part of combination therapies, including immunotherapy, and integrating them with current standard treatments will be crucial. Additionally, identifying predictive biomarkers for combination therapy responses remains a key area of interest.

## 3 TARGETING MUTATED IDH1 AND IDH2: AT THE CROSSROADS OF METABOLIC AND EPIGENETIC DYSREGULATION

Initially identified in the rare neurological disorder 2‐hydroxyglutaric aciduria, D‐2‐Hydroxyglutarate (2‐HG) gained significant attention in 2009, when genome‐wide sequencing of gliomas and acute myeloid leukemia (AML), coupled with groundbreaking research on isocitrate dehydrogenases (IDH), uncovered its synthesis by mutated IDH1 (mIDH1) or IDH2 (mIDH2) enzymes.^159^, ^160^, ^161^, ^162^, ^163^, ^164^ mIDH1/mIDH2 were subsequently discovered in various malignancies such as gliomas (mIDH1 in 80% of low‐grade gliomas and secondary glioblastoma), AML (mIDH1 8%, mIDH2 12%), myelodysplastic (3%–4% mIDH1, 6% mIDH2) and myeloproliferative (1%–4%, mIDH2 more common) neoplasia, T‐cell acute lymphoblastic leukemia (mIDH1 2%–3%, mIDH2 5%), cholangiocarcinoma (mIDH1 9%–12%, mIDH2 5%) and chondrosarcoma (mIDH1 40%, mIDH2 12%).^2^, ^3^, ^165^, ^166^, ^167^, ^168^, ^169^, ^170^, ^171^, ^172^, ^173^, ^174^, ^175^, ^176^

Under physiological conditions, IDH1 and IDH2 convert isocitrate to alpha‐ketoglutarate (α‐KG) and generate NADPH in this process. Mutations at conserved arginine residues‐R132 in mIDH1 and R140 or R172 in mIDH2‐impair the normal catalytic function of IDH1/IDH2 and preferentially produce 2‐HG instead of α‐KG. This phenomenon is known as “neomorphic” activity.^162^, ^163^, ^164^ Functionally, 2‐HG exerts its effects primarily through a competitive inhibition of α‐ketoglutarate‐dependent dioxygenases.^163^ This inhibitory action points to a remarkable intersection between metabolic and epigenetic dysregulation in cancer. From the metabolic perspective, 2‐HG inhibits HIF prolyl hydroxylases, which leads to elevated HIF‐1α levels and a state of persistent pseudohypoxia. Moreover, 2‐HG inhibits cytochrome c oxidase in the mitochondrial electron transport chain and lowers the threshold for apoptosis in response to BCL‐2 inhibition.^177^, ^178^, ^179^, ^180^, ^181^, ^182^ These actions preferentially preserve the survival of IDH‐mutated cancer cells. However, at the same time, the depletion of α‐ketoglutarate inactivates TET enzymes and JmjC‐domain histone demethylases, causing epigenetic dysregulation through widespread CpG/DNA methylation.^183^, ^184^, ^185^, ^186^, ^187^, ^188^, ^189^, ^190^ This silences tumor suppressor genes but also aberrantly activates oncogenes. For example, mIDH1 gliomas exhibit hypermethylation at insulator‐associated CTCF binding sites, which disrupts insulator function and allows a constitutive enhancer to interact with the PDGFRA oncogene.^189^

Of note, IDH1 is located in the cytosol, while IDH2 is present in the mitochondria. Compartmental differences in substrate availability (such as variations in αKG and NADPH between the cytosol and mitochondria) might create distinct metabolic and subsequent epigenetic profiles.^191^, ^192^, ^193^ For instance, mIDH1 but not mIDH2 AML shows depletion of monoglycerides and lysophospholipids, despite similar 2‐HG elevations.^193^ This suggests putative unique vulnerabilities between these mutant enzymes.

### 3.1 Clinical relevance for therapeutic targeting for mIDH1 and mIDH2

Since the discovery of mutant IDH1/2, four compounds designed to reduce 2‐HG production have received regulatory approval. Ivosidenib (AG‐120), a selective inhibitor of mIDH1, received FDA approval in 2017 for relapsed/refractory AML in elderly patients or those ineligible for intensive chemotherapy.^194^, ^195^, ^196^ This was based on the AG120‐C‐001 Phase 1 trial (NCT02074839), which demonstrated significant clinical efficacy with an overall response rate (ORR) of 41.6%.^195^, ^196^ By October 2023, based on the same trial, Ivosidenib also gained FDA approval for the treatment of IDH1‐mutated myelodysplastic syndromes, demonstrating a complete remission (CR) rate of 38.9%.^197^

The anticipated common impact in reducing hypermethylated DNA and histones suggests a synergistic potential between mIDH inhibitors and hypomethylating agents like 5‐azacytidine (Aza).^198^ Promising results from an early‐phase trial (NCT02677922) in AML led to the Phase 3 AGILE trial (NCT03173248), which compared Ivosidenib + Aza to placebo + Aza in newly diagnosed patients.^199^, ^200^ Here, at a median follow‐up of 12.4 months, event‐free survival (EFS) was significantly longer in the Ivosidenib‐Aza group (HR 0.33), with probabilities of 40% at 6 months and 37% at 12 months, compared to 20% and 12%, respectively, in the placebo‐Aza group, establishing Ivosidenib‐Aza as a new standard of care for AML patients with mIDH1 ineligible for intensive therapy (approved in both the USA and Europe in 2023).^200^

Beyond hematologic malignancies, Ivosidenib is approved for IDH1‐mutated advanced/metastatic cholangiocarcinoma. In the Phase III ClarIDHy trial (NCT02989857), Ivosidenib significantly improved progression‐free survival (PFS) versus placebo (2.7 vs. 1.4 months), with a median overall survival (OS) of 10.3 months compared to 5.1 months in the placebo group after adjusting for crossover.^201^

Enasidenib (AG‐221), a targeted inhibitor of mIDH2, received FDA approval for relapsed/refractory AML in 2017 based on results from the AG221‐C‐001 trial (NCT01915498), where a composite CR (cCR) rate of 28.6% was achieved in 214 patients.^202^, ^203^ In a subsequent Phase 3 trial (NCT02577406), Enasidenib was compared to conventional care regimens in elderly patients with relapsed/refractory mIDH2 AML. While the primary OS endpoint (6.5 vs. 6.2 months) was not met due to early dropouts and the availability of Enasidenib outside the trial, the drug showed significant improvements in all secondary efficacy endpoints.^204^ In a Phase 2/1b Beat AML substudy (NCT03013998), Enasidenib was further evaluated in patients with treatment‐naive AML and mIDH2. The cCR rate was 46%. For patients who did not achieve remission on Enasidenib alone, combination therapy with Aza led to a cCR rate of 41%, supporting the putative clinical utility of this combination.^205^ In a separate phase 1b/2 trial (NCT02677922), Enasidenib plus Aza achieved a 74% ORR compared to 36% with Aza alone.^206^ However, it remains to be demonstrated whether this combination will reach clinical approval.

Olutasidenib (FT‐2102), another selective mIDH1 inhibitor, received FDA approval in December 2022 for relapsed/refractory AML. This compound binds to a hydrophobic pocket near the IDH1 active site in a 2:1 stoichiometric ratio and may overcome resistance to other mIDH1 inhibitors.^207^, ^208^ In a phase 1/2 trial (NCT02719574), Olutasidenib achieved an ORR of 38% as monotherapy and 56% in combination with Aza.^209^ In the Phase 2 interim analysis of 153 IDH1 inhibitor‐naive patients, the cCR rate was 35%, with a median OS of 11.6 months and a cCR duration of 25.9 months.^210^

Vorasidenib (AG‐881), a dual inhibitor of mutant IDH1 and IDH2 designed to penetrate the blood–brain barrier, recently received FDA approval for treating Grade 2 astrocytoma or oligodendroglioma, with European approval expected by early 2025.^211^, ^212^ In the INDIGO trial (NCT04164901), 331 patients with IDH1 or IDH2‐mutant tumors were randomized to receive Vorasidenib or placebo. Vorasidenib significantly delayed disease progression, with a hazard ratio of 0.39 for PFS compared to placebo. The median time to the next intervention had not been reached in the Vorasidenib group, while it was 17.8 months in the placebo group.^212^

### 3.2 Special clinical considerations for mIDH inhibition

#### 3.2.1 Minimal residual disease

Monitoring IDH mutations for Minimal Residual Disease (MRD) has gained traction in AML, especially with advancements in next‐generation sequencing (NGS)‐ or digital‐PCR‐based MRD monitoring methods. However, it appears that mIDH clearance (NGS‐MRD <0.1%) is not an absolute requirement for improved survival.^213^, ^214^, ^215^ As such, the clinical utility of MRD for mIDH1/mIDH2 remains experimental and requires validation before it can inform treatment decisions.

#### 3.2.2 Differentiation syndrome

In AML, IDH inhibitors reverse the differentiation block that defines the disease.^183^, ^185^, ^190^ However, this process may trigger a synchronized differentiation, leading to differentiation syndromes (DS) (10%–20% frequency under mIDH1/2 inhibition).^216^, ^217^, ^218^ DS are potentially life‐threatening complications characterized by fever, pulmonary infiltrates/respiratory distress, weight gain, peripheral edema/pleuro‐pericardial effusions, hypotension, and renal failure.^61^ Therapeutic intervention typically includes respiratory support, corticosteroids, and, in severe cases, temporary discontinuation of the IDH inhibitor.^218^, ^219^, ^220^ While much knowledge on managing DS comes from experience with ATRA/ATO‐induced DS in acute promyelocytic leukemia (APL), key differences exist in mIDH1/2‐related DS.^219^ Unlike the rapid onset typically seen in APL, DS in AML patients treated with mIDH inhibitors tend to occur later (weeks/months after treatment initiation).^216^, ^217^, ^218^ Notably, DS often coincide with clinical responses—marked by falling blast counts in the blood and the emergence of mature myeloid cells.^216^, ^217^, ^218^

### 3.3 Conclusions and future directions

Mutated IDH1/IDH2 enzymes represent a prime example of the intersection between metabolic and epigenetic dysregulation in cancer biology. The recent approval of targeted inhibitors for both hematologic and solid malignancies marks a significant advancement in precision oncology and offers new hope in challenging cancers. However, this progress likely represents only the initial phase. Ongoing studies that explore novel combination therapies, including their integration into intensive treatment protocols, hold promise for further improvements of response/remission rates in these difficult‐to‐treat cancers.

## 4 ONCOGENIC SWI/SNF COMPLEXES: MECHANISTIC DISCOVERIES AND THERAPEUTIC OPPORTUNITIES

### 4.1 SWI/SNF complex composition and function

The family of SWI/SNF (Switching‐defective/Sucrose‐Non‐Fermenting, in mammals referred to as BAF BRG1‐ or BRM‐associated‐factors) chromatin remodeling complexes is ubiquitously expressed and consists of multi‐subunit complexes that differ in their compositions, containing a BAF‐core and an ATPase module as shared building blocks and several subcomplex‐specific proteins.^221^, ^222^ The core ATPase can be either SMARCA4 (BRG1) or SMARCA2 (BRM) bound in a functional module together with beta‐actin, SS18, and BCL7 as a shared functional unit in all BAF complexes.^222^ To date, three unique BAF‐complexes have been defined: the canonical BAF (BAF/cBAF)‐, the polybromo‐associated BAF (PBAF)‐ and the non‐canonical BAF (ncBAF)‐complexes. Aside from the ATPase module, all of them share an initial BAF core consisting of SMARCC1/2 and SMARCD1/2/3 proteins. After incorporation of SMARCB1 and SMARCE1, this BAF‐core is the backbone of cBAF and PBAF complexes. Together with ARID1A/B, DPF1/2/3, and the ATPase module, the cBAF complex is formed based on the BAF core. When associated with ARID2, BRD7, PHF1, PB1, and the ATPase module, the alternative PBAF complex is formed. In contrast, the ncBAF complex is based on only the initial BAF core (SMARCC/SMARCD) and incorporates GLTSCR1 and BRD9 together with the ATPase module.^222^ These three BAF‐complexes are critical mediators of histone turnover, particularly at transcriptionally active sites in the genome, including promoters and enhancers, with redundant and non‐redundant functions and implications in cancer development.^223^ BAF complexes catalyze the remodeling of nucleosomes and are therefore critical for the temporal and spatial regulation of chromatin accessibility.^224^, ^225^ Specifically, BAF complexes, as mammalian homologues of Drosophila Trithorax, have a critical role in opposing Polycomb‐complex function, particularly at bivalent promoters and enhancer elements.^226^ If BAF‐complex function is perturbed (e.g., by mutations), the balance between activation and repression of those sites is disrupted, and polycomb‐mediated repression becomes dominant and silences these bivalent sites, leading to aberrant gene regulation. As explained in section 1, this phenomenon is the mechanistic explanation for why SWI/SNF mutant cancers like rhabdoid tumors have a strong addiction to EZH2, the catalytic subunit of the PRC2 complex.

Due to their complex and critical roles for balancing chromatin states at gene regulatory elements, SWI/SNF‐complexes have diverse functional implications in cellular differentiation, cell cycle regulation, and DNA‐damage repair.^227^, ^228^, ^229^, ^230^ Cancer genome sequencing studies showed that mutations of SWI/SNF‐complex subunits occurred in approximately 25% of human malignancies.^231^ Therefore, targeting oncogenic functions of SWI/SNF‐complexes or leveraging genotype‐related synthetic‐lethality has been a focus in epigenetic drug development during the recent year.^232^, ^233^

### 4.2 Therapeutic targeting of SWI/SNF‐function in cancers

To understand the mechanistic concepts behind the targeting of SWI/SNF‐related functions in cancer, it is important to remember the fact that these protein complexes can act as oncogenes or tumor suppressors depending on the cellular context.

Many cancers, particularly those driven by transcription‐related oncogenic mechanisms, heavily rely on SWI/SNF‐complexes, and global inhibition of the function of this machinery might have a therapeutic index. The concept of global SWI/SNF‐inhibition has been explored preclinically, particularly in the field of myeloid blood cancers.^234^, ^235^, ^236^, ^237^ A dual ATPase inhibitor of the common catalytic subunits SMARCA2 and SMARCA4 (FHD‐286) has been evaluated in early phase clinical trials (Table 4). Preliminary results from the first‐in‐human phase I study using FHD‐286 (NCT04891757) showed marked toxicity, with 85% of patients experiencing treatment‐related adverse events (TRAE), including 50% having a grade 3 or higher TRAE.^238^ Notably, differentiation induction was noted in some patients, including clinically significant differentiation syndrome, providing some evidence for on‐target activity. In contrast to the concept of inhibiting global SWI/SNF activity in cancers dependent on trithorax homologues, these chromatin remodelling complexes are critical tumor suppressors. Most cancer‐driving mutations in SWI/SNF family members lead to a loss‐of‐function of the affected protein and therefore a compromised function of the affected BAF‐subcomplex. Due to compensatory mechanisms in BAF‐complex assembly, these loss‐of‐function somatic mutations within the cancer cells may lead to cancer‐specific synthetic lethality that can be leveraged for treatment approaches with a greater therapeutic index. For example, SMARCA4 mutant cancers become highly addicted to SMARCA2 function since the enzyme compensates for functional SMARCA4 loss in the ATPase module across all BAF complexes. Therefore, the selective depletion of SMARCA2 is particularly toxic for the respective cancer cells, while non‐malignant normal cells can compensate for SMARCA2 loss via SMARCA4 function.^239^, ^240^ The fact that the enzymatic pockets of SMARCA4 and SMARCA2 are strongly conserved and highly similar in structure has made it difficult to establish selective enzymatic inhibitors. The bromodomains via which both proteins bind to acetylated histones have more unique structural features, which allowed the design of selective bromodomain inhibitors.^241^ Unfortunately, those inhibitors, despite their ability to penetrate cells and potently block SMARCA2 bromodomains, did not exert antiproliferative effects in cancer cells. Nevertheless, the development of these lead compounds was a true milestone since recent advances in drug development have now allowed us to use these compounds as warheads for highly SMARCA2‐selective targeted protein degraders (PROTACs).^242^ The first SMARCA2 PROTAC, PRT3789, has now entered early‐phase clinical trials in SMARCA4 mutant solid cancers; an early report demonstrates a favourable tolerability profile and first responses even in the monotherapy setting.^243^

**TABLE 4:** Therapeutics that directly target components of the SWI/SNF complex.

Interven‐tional agents	Disease (setting)	Study phase	ID	Patients enrolled	Status	Reference
FHD‐286 (SMARCA4/−2‐ATPase‐i)	Metastatic UVM	I	NCT04879017	73	Terminated	Foghorn theraupeutics^277^
FHD‐286 (SMARCA4−/2‐ATPase‐i)	R/R AML, R/R MDS, and R/R CMML not in blast crisis	I	NCT04891757	40	Terminated	ASH 2023^238^
FHD‐909 (SMARCA2‐ATPase‐i)	SMARCA4‐mutant cancers				Recruiting	Foghorn Therapeutics, 10.10.2024
PRT3789 (SMARCA2 degrader) ± docetaxel	SMARCA4 mutant advanced or metastatic solid tumors	I	NCT05639751	n.a. *(e: 186)*	Recruiting	Annals of Oncology^243^
FHD‐609 (BRD9 degrader)	Advanced synovial sarcoma or advanced SMARCB1‐loss tumors	I	NCT04965753	55	Terminated^o^	
CFT8634 (BRD9 degrader)	Locally advanced or metastatic synovial sarcoma and other	I/II	NCT05355753	50	Terminated	C4 Therapeutics,^278^ clinicaltrials.gov^279^

*Note*: Therapeutics that directly target components of the SWI/SNF complex.

Abbreviations: AML, acute myeloid leukemia; CMML, chronic myelomonocytic leukemia; MDS, myelodysplastic syndrome; n. a., not available; R/R, relapsed/refractory; TKI, tyrosine kinase inhibitor; UVM, uveal melanoma; *e*, estimated; ^Δ^, pending approval of cohort expansion; ^o^, sponsor decision.

Another setting in which compensatory mechanisms within BAF‐complexes lead to synthetic lethality that can be leveraged for targeted cancer therapies is cancers with SMARCB1 mutations, found in a variety of mesenchymal neoplasms including rhabdoid tumors and other sarcomas.^244^ SMARCB1 is a component of the BAF‐core module in cBAF and PBAF but not in ncBAF complexes and is required for polycomb‐antagonism at a critical subset of regulatory regions in the genome.^222^, ^226^ Similarly, SS18 fusion proteins in synovial sarcoma impair BAF complex function and create a selective vulnerability for ncBAF.^245^, ^246^ In the context of SMARCB1 mutations or SS18 fusion proteins, ncBAF complex activity is increased and compensates for the loss of cBAF and PBAF function; hence, SMARCB1 mutant tumor cells are explicitly vulnerable to perturbation of ncBAF activity.^247^, ^248^, ^249^ A critical and unique component of ncBAF is the bromodomain protein BRD9.^222^ Due to the absence of an enzymatic moiety, the bromodomain of this protein has been identified as the most attractive target for drug development. Similar to SMARCA2, bromodomain inhibition only showed modest functional activity in preclinical cancer models.^250^ Again, PROTACs using BRD9‐selective bromodomain binders as warheads have shown relevant preclinical activity and are currently explored in early‐phase clinical trials in cancers with disturbed cBAF/PBAF function.^251^, ^252^, ^253^, ^254^ Two BRD9 degraders, FHD‐609 and CFT8634, were so far tested in clinical trials and showed an overall favorable safety profile (Table 4); yet, the clinical development is currently halted due to limited activity in the patient population investigated.

Aside from mutation‐specific vulnerabilities within BAF complexes themselves, cancers with mutations in the most frequently affected BAF subunits ARID1A, SMARCA4/2, and SMARCB1^255^ have several other synthetic lethal interactions that can be leveraged therapeutically. Table 5 gives an overview of ongoing clinical trials investigating interventional agents on cancers with mutated subunits of the SWI/SNF complex. Numerous different substance classes are currently being evaluated in early‐phase clinical trials. The tested agents include EZH2 inhibitors that aim to exploit the above‐described dependency of cancers with SWI/SNF loss‐of‐function mutations on the PRC2 complex. Furthermore, SMARCA4 loss has been described to cause Cyclin D1 deficiency and consequent synthetic lethal dependency on CDK4/6.^256^ Therefore, CDK4/6 as well as Aurora A kinase inhibition has been probed as therapeutic interventions in SWI/SNF mutant cancers (Table 5). Moreover, BAF‐complexes cooperate with the DNA repair machinery to cope with DNA damage. Therefore, targeting of PARP or ATR has also been described to be synthetic lethal in the presence of SWI/SNF complex mutations.^257^, ^258^ Increased sensitivity for immune‐checkpoint‐inhibition (ICI) has also been documented in various cancers bearing SWI/SNF mutations, although the exact mechanisms remain elusive.^259^, ^260^

**TABLE 5:** Interventional clinical trials involving cancers with mutations of SWI/SNF subunits.

Mutated SWI/SNF subunit	Interventional agents (target)	Disease (setting)	Study phase	ClinicalTrial.gov ID	Patients enrolled	Status	Reference
*ARID1A*	Tazemetostat (EZH2i)	Advanced or metastatic solid tumors	II	NCT05023655	n.a. *(e: 40)*	Recruiting	
*ARID1A*	Olaparib (PARPi)	Metastatic biliary tract cancer with mutations in DNA repair genes	II	NCT04042831	n.a. *(e: 36)*	Recruiting	
*ARID1A*	Niraparib (PARPi)	UVM, mesothelioma, CCRCC or CCA with mutations in DNA repair genes	II	NCT03207347	37	Terminated	ASCO 2022^280^
*ARID1A/ARID2*	Niraparib (PARPi)	Advanced, metastatic melanoma	II	NCT03925350	14	Terminated	ESMO 2024^281^
*ARID1A*	Olaparib (PARPi) ± Capivasertib (ATRi)	Solid tumors with mutations in DNA repair genes	II	NCT02576444	67	Terminated	clinicaltrials.gov^282^
*ARID1A*	Bevacizumab (VEGF‐AK) ± Niraparib (PARPi)	Recurrent endometrial and OC	II	NCT05523440	n.a. *(e: 92)*	Recruiting	
*ARID1A*	Ceralasertib (ATRi) ± Olaparib (PARPi) ± Durvalumab (ICI)	Relapsed gynecological cancers	II	NCT04065269	29	Recruiting	ESMO 2023^283^
*ARID1A*	Berzosertib (ATRi)	Advanced solid tumors	II	NCT03718091	29	Completed	clinicaltrials.gov^284^
*ARID1A*	Tuvusertib (ATRi) + ZEN‐3694 (pan‐BETi)	Recurrent endometrial and OC	I	NCT05950464	n.a. *(e: 60)*	Recruiting	
*ARID1A*	Tuvusertib (ATRi) + Avelumab (ICI)	Endometrial cancers with prior immunotherapy	II	NCT06518564	n.a. *(e: 25)*	Not yet recruiting	
*ARID1A*	PLX2853 (BET/BRD4i) + Carboplatin	Platinum‐resistant epithelial OC	I/II	NCT04493619	37	Terminated	AACR J. Vol. 8/2023^285^
*ARID1A*	Dasatinib (TKI) + Toripalimab (ICI)	Advanced NSCLC	II	NCT04284202	n.a. *(e: 30)*	Unknown status	
*ARID1A*	Dasatinib (TKI)	R/R fallopian tube, endometrial or peritoneal and OC	II	NCT02059265	28	Terminated	Gynecol. Oncol. Vol. 176/2023^286^
*ARID1A*	Nivolumab (ICI)	Metastatic or unresectable solid tumors	II	NCT04957615	n.a. *(e: 30)*	Recruiting	
*ARID1A*	Nivolumab (ICI)	Metastatic urothelial cancer	II	NCT04953104	n.a. *(e: 30)*	Recruiting	
*ARID1A*	Tremelimumab (ICI) + Durvalumab (ICI) + Belinostat (HDACi)	Urothelial carcinoma	I	NCT05154994	*9*	Suspended^δ^	
*ARID1A*	JAB‐2485 (Aurora A kinase i)	Advanced solid tumors	I/II	NCT05490472	n.a. *(e: 102)*	Recruiting	
*ARID1A*	NXP800 (HSF1 pathway i)	Clear cell and endometrioid OC	I	NCT05226507	n.a. *(e: 61)*	Recruiting	
*SMARCB1* or *SMARACA4*	Tazemetostat (EZH2i)	R/R pediatric brain tumors, solid tumors, NHL, histiocytic disorders	II	NCT03213665	20	Active, not recruiting	JNCI Vol.115/2023^287^
*SMARCB1* or *SMARACA4*	Tazemetostat (EZH2i)	Pediatric R/R rhabdoid tumor, sarcoma, chordoma or other	I	NCT02601937	109	Completed	ASCO 2022^288^
*SMARCB1* or *SMARACA4*	Tazemetostat (EZH2i) + Nivolumab/Ipilimumab (ICI)	Rhabdoid tumor, sarcoma, chordoma or other	I/II	NCT05407441	n.a. *(e: 49)*	Recruiting	
*SMARCB1* or *SMARACA4*	Tazemetostat (EZH2i) + Doxorubicin	Adult soft tissue sarcoma	II	NCT02601950	62	Completed	Lancet Oncol. Vol. 21/2020^289^
*SMARCB1* or *SMARACA4*	Alisertib (Aurora A‐kinase i) ± chemotherapy	pediatric rhabdoid tumors	II	NCT02114229	30	Active, not recruiting	Neuro‐Oncol. Vol. 25/2023^290^
*SMARCB1* or *SMARACA4*	Tiragolumab (ICI) + Atezolizumab (ICI)	R/R RMC, rhabdoid tumors, chordoma, sarcoma or other	I/II	NCT05286801	n.a. *(e: 86)*	Recruiting	
*SMARCB1*	Tazemetostat (EZH2i) + Docetaxel, Cisplatin, 5‐FU	Locally advanced non‐metastatic sinonasal carcinoma	II	NCT05151588	n.a. *(e: 30)*	Not yet recruiting	
*SMARCB1*	Ubamatamab (MUC16xCD3 bispecific ab) ± Cemiplimab (ICI)	RMC and epithelioid sarcoma	II	NCT06444880	n.a. *(e: 40)*	Not yet recruiting	
*SMARCA2* and/or *SMARCA4*	Ibrutinib (TKI), Rituximab (anti‐CD20‐ab), Venetoclax (BCL2i) and Navitoclax (BCL2i)	MCL high risk	II	NCT05864742	n.a. *(e: 40)*	Active, not recruiting	

*Note*: Recent interventional clinical trials that investigate therapeutic effects of different agents on cancers with mutated SWI/SNF components.

Abbreviations: ab, antibody; CCA, cholangiocarcinoma; CCRCC, clear cell renal cell carcinoma; i, inhibitor; ICI, immune checkpoint inhibitor; MCL, mantle cell lymphoma; NHL, non‐Hodgkin lymphoma; OC, ovarian cancer; RMC, renal medullary carcinoma; R/R, relapsed/refractory; TKI, tyrosine kinase inhibitor; UVM, uveal melanoma. n. a., not available; *e*, estimated; ^Δ^, pending approval of cohort expansion; ^o^, sponsor decision.

Although significant efforts have been made in drug development and clinical testing on these hard‐to‐treat cancer types, most of the presented trials did not meet the pre‐specified response criteria. Nevertheless, moderate responses and anti‐tumor activity, disease stabilization, and clinical benefits are described in a subset of patients. Further evaluation of these therapeutic strategies and biomarker development for better stratification of patients is certainly needed.

### 4.3 Conclusions and future directions

The SWI/SNF (BAF) chromatin remodeling complexes are critical regulators of chromatin accessibility and transcription, composed of three alternative complex assemblies: cBAF, PBAF, and ncBAF. These complexes share a core structure but differ in subunit composition with redundant and non‐redundant functions. Mutations in SWI/SNF subunits are found in 25% of human cancers, disrupting chromatin dynamics and driving oncogenesis. Depending on the context, SWI/SNF complexes can act as oncogenes or tumor suppressors, making them attractive therapeutic targets. Global inhibition of this protein complex family, such as with the dual ATPase inhibitor FHD‐286, showed limited efficacy and substantial toxicity in early clinical trials. Exploiting synthetic lethal interactions caused by specific mutations offers more promise, as in SMARCA4‐mutant cancers reliant on SMARCA2 or SMARCB1‐mutant tumors vulnerable to ncBAF‐targeting. Advances like the PROTAC technology have enabled the development of targeted degraders, such as PRT3789 and BRD9‐specific agents, showing early potential in clinical trials.

Combination therapies targeting other synthetic lethal vulnerabilities, such as with EZH2, CDK4/6, or PARP inhibitors, are also being investigated, alongside immune checkpoint inhibitors. However, clinical outcomes remain modest, with responses seen only in a subset of patients. Continued efforts in biomarker development and refined therapeutic strategies are essential to improve treatment for SWI/SNF‐mutant cancers.

## 5 OUTLOOK AND PERSPECTIVES

The FDA‐approvals of IDH, Menin, and EZH2 inhibitors have demonstrated that true targeted chromatin therapy is feasible and can lead to substantial clinical benefits in patients. Of note, in contrast to broader and more unselective epigenetic therapy approaches like demethylating agents or HDAC‐inhibitors, those therapies are overall well tolerated and have a relatively broad therapeutic windows. Nevertheless, these examples have also demonstrated that rigorous molecular studies, functional genomics, drug‐screening approaches, and preclinical studies are required to define precise disease entities that may respond to these highly selective treatments. A detailed understanding of underlying oncogenic mechanisms becomes more and more important to allocate patients to the right substances. The fact that sophisticated genomic profiling approaches like genome‐, exome‐ or targeted panel‐sequencing, become more accessible and are now frequently applied in clinical routine across several cancer entities will fuel future developments and help to define new patient populations that may benefit from targeted chromatin therapies. In this review, we have largely focused on the implications of epigenetic inhibitors on tumor‐intrinsic functions. It is worth noting, though, that the impact of those drugs on other cells, particularly the immune microenvironment, is not to be underestimated. Importantly, most of the selective chromatin‐modifying therapies described in this manuscript do not impair global immunological functions. In contrast, some even seem to improve immune‐recognition and clearance of cancer cells. A common mechanism of action seems to be the de‐repression of repetitive genomic elements in cancer cells leading to increased abundance of double‐stranded RNA, higher immunogenicity, and improved clearance by CD8+ T‐cells.^99^, ^261^ This phenomenon has so far been observed for Menin as well as for EZH2 inhibitors.

A major flaw that hinders this development in many areas and complicates the design of more unbiased clinical trials for almost all chromatin‐targeting compounds is the lack of clinically amendable response biomarkers. In the experimental setting, chromatin immunoprecipitation and chromatin accessibility assays, as well as transcriptome analysis of defined cell populations, can be used to determine biologically relevant responses; yet none of these assays can currently be used in clinical routine. A good example of this problem is the clinical management of patients under Menin inhibitor treatment. Even though being approved by the FDA as a highly effective treatment option for patients with relapsed or refractory leukemia with KMT2A‐rearrangements or NPM1‐mutations, roughly 50% of patients show no clinical response.^65^, ^262^ Although mutations in the MEN1‐gene have been linked to acquired Menin inhibitor resistance, no genetic or non‐genetic causes of up‐front resistance to treatment could be defined so far. An even more drastic situation exists in patients with epitheloid cancers treated with EZH2‐inhibitors. Here, the overall response rate is only around 15%,^15^ but a relevant fraction of those responders shows deep and long‐lasting disease control. Therefore, a critical goal for the near future is the establishment of clinically amendable response biomarkers to improve the stratification of patients for clinical trials or approved substance use. This will be essential to increase efficacy across the treated patient populations. Furthermore, a better patient stratification will also be important for an economic future use of these substances, since ineffective prescriptions would be a significant burden for health care systems.

An important topic related to these considerations is the establishment of effective combination therapy approaches. In many cancers, the future use of chromatin targeting therapeutics will be in combination with chemotherapy, apoptosis‐inducers, or other small molecular inhibitors or degraders. It is worth noting that these combination therapies will make it even more challenging to disentangle the activity of the individual drugs and to identify biomarkers that predict the added advantage of the individual components of a respective regimen. Due to medical, legal, and regulatory complexities around clinical trials probing multiple combinations of targeted agents, consequent harmonization and exchange of datasets will be critical to drive timely discoveries and guide clinicians when making combination therapy decisions. The availability of modern artificial intelligence tools will make the identification of so far unknown patterns in such datasets possible, but so far, clinical datapoints in the space of chromatin therapeutics are limited, and many data sources are not available in a structure that is directly amendable to machine learning.

It is to be expected that the spectrum of aberrant epigenetic functions against which small‐molecule compounds are available will greatly expand in the next decade. In this manuscript, we have largely focused on molecules that interfere with post‐translational modifications of histones and/or chromatin structure, yet several molecules interfering with transcription or chromatin conformation in other ways have been developed or are currently under development. Two important classes of drugs that have allowed targeting of previously “undruggable” targets are protein–protein interaction inhibitors (like Menin inhibitors) and targeted protein degraders. In the field of epigenetics, particularly Bromodomain‐inhibitors have raised attention. Bromodomains are protein domains with which so‐called “chromatin‐reader” proteins bind to acetylated histones. The spectrum of these Bromodomain proteins is wide and a diverse set of chemical probes allows for selective or non‐selective blockade of these protein domains enabling the blocking of the interaction of such reader proteins with acetylated chromatin.^263^, ^264^, ^265^ One of the most well‐known examples is the “Bromodomain 4” (BRD4) protein which binds to H3K27ac and is an essential co‐activator of transcription.^266^, ^267^ As of now, several BRD4 inhibitors have been developed and entered the clinical pipeline, but in most instances, response rates are limited and adverse events frequent.^268^ Nevertheless, further development in the field of Bromodomain inhibitors holds promise for the development of highly selective compounds to interrogate acetyl‐reader binding on chromatin in specific disease settings in the future.

Another exciting area of drug development is the targeted degradation of specific transcription factors (TFs). Mechanistic studies with so‐called “Immunomodulatory drugs” (IMIDs), like thalidomide, lenalidomide, or pomalidomide, which have been routinely used in the treatment of multiple myeloma for many years, have demonstrated that these drugs can selectively degrade specific zinc‐finger transcription factors.^269^, ^270^ Large libraries of chemically modified IMIDs have been generated and have demonstrated that the potency and selectivity against individual TFs can greatly be improved.^271^, ^272^, ^273^ One example of the clinical success of this approach is the novel IMID Mezigdomide, which is almost purely selective for the degradation of the IKZF1 TF and has a much higher potency in doing so compared to lenalidomide or pomalidomide.^274^, ^275^ Further screening and structural optimization of such libraries are expected to yield more novel IMIDs with explicit selectivity for certain zinc‐finger TFs.

In conclusion, targeted chromatin therapy is now feasible, and the first compounds have already received approval from regulatory agencies. The field of epigenetic therapeutics is rapidly evolving, and it is to be expected that more substances targeting epigenetic writers, erasers, readers, or transcription factors will be available for the treatment of cancer patients in the years to come.

## AUTHOR CONTRIBUTIONS

**Florian Perner:** Writing – original draft; writing – review and editing. **Tobias Berg:** Writing – original draft; writing – review and editing. **Daniel Sasca:** Writing – original draft; writing – review and editing. **Sophie‐Luise Mersiowsky:** Writing – original draft; writing – review and editing. **Jayant Y. Gadrey:** Writing – original draft; writing – review and editing. **Johanna Thomas:** Project administration and editing. **Michael W. M. Kühn:** Writing – original draft; writing – review and editing. **Michael Lübbert:** Conceptualization; writing – original draft; writing – review and editing; visualization; project administration; supervision; resources.

## CONFLICT OF INTEREST STATEMENT

Florian Perner received travel support from Syndax Pharmaceuticals and CHARM Therapeutics. Tobias Berg received research funding from Imago Biosciences (a subsidiary of Merck) and honoraria from AVIR Pharma, Jazz Pharmaceuticals, Bristol Myers Squibb, and Riemser Pharma GmbH, as well as travel funding from Incyte, Abbvie, Astellas, Alexion, and Celgene. Michael Lübbert received public funding support from the German Research Foundation (DFG) for the DECIDER‐2 clinical trial, support from Cheplapharm for study drug supply, Janssen provided research support to the institution (University Medical Center Freiburg) and received funding from the DFG (FOR 2674, A05, A09, Medep‐CRC992, DKTK Freiburg, Deutsche José Carreras Leukämie‐Stiftung e. V. R14/25). Daniel Sasca received honoraria from Abbvie, AstraZeneca, Gilead, Blueprint, has been on the advisory board for Novartis, and received travel support from Abbvie, AOP Health, and Novartis. Michael W. M. Kühn has potential financial conflicts of interest with Abbvie, Blueprint, BMS/Celgene, Gilead, Johnson & Johnson, Jazz, Kura Oncology, Pfizer, Servier, and Syndax. All other authors have no relevant conflicts of interest to disclose.

## References

[1] Husmann D , Gozani O . Histone lysine methyltransferases in biology and disease. Nat Struct Mol Biol. 2019;26(10):880‐889. doi:10.1038/s41594-019-0298-7 31582846 PMC6951022

[2] Murn J , Shi Y . The winding path of protein methylation research: milestones and new frontiers. Nat Rev Mol Cell Biol. 2017;18(8):517‐527. doi:10.1038/nrm.2017.35 28512349

[3] Herz HM , Garruss A , Shilatifard A . SET for life: biochemical activities and biological functions of SET domain‐containing proteins. Trends Biochem Sci. 2013;38(12):621‐639. doi:10.1016/j.tibs.2013.09.004 24148750 PMC3941473

[4] Morera L , Lübbert M , Jung M . Targeting histone methyltransferases and demethylases in clinical trials for cancer therapy. Clin Epigenetics. 2016;8(1):57. doi:10.1186/s13148-016-0223-4 27222667 PMC4877953

[5] Petrossian TC , Clarke SG . Uncovering the human Methyltransferasome. Mol Cell Proteomics. 2011;10(1):M110.000976. doi:10.1074/mcp.M110.000976 PMC3013446 20930037

[6] Wang MY , Liow P , Guzman MIT , Qi J . Exploring methods of targeting histone methyltransferases and their applications in cancer therapeutics. ACS Chem Biol. 2022;17(4):744‐755. doi:10.1021/acschembio.2c00062 35363464 PMC9336197

[7] Rugo HS , Jacobs I , Sharma S , et al. The promise for histone methyltransferase inhibitors for epigenetic therapy in clinical oncology: a narrative review. Adv Ther. 2020;37(7):3059‐3082. doi:10.1007/s12325-020-01379-x 32445185 PMC7467409

[8] Perner F , Armstrong SA . Targeting chromatin complexes in myeloid malignancies and beyond: from basic mechanisms to clinical innovation. Cells. 2020;9(12):2721. doi:10.3390/cells9122721 33371192 PMC7767226

[9] Piunti A , Shilatifard A . Epigenetic balance of gene expression by Polycomb and COMPASS families. Science. 2016;352(6290):aad9780. doi:10.1126/science.aad9780 27257261

[10] Schuettengruber B , Bourbon HM , Di Croce L , Cavalli G . Genome regulation by Polycomb and Trithorax: 70 years and counting. Cell. 2017;171(1):34‐57. doi:10.1016/j.cell.2017.08.002 28938122

[11] Duan R , Du W , Guo W . EZH2: a novel target for cancer treatment. J Hematol OncolJ Hematol Oncol. 2020;13(1):104. doi:10.1186/s13045-020-00937-8 32723346 PMC7385862

[12] Cyrus S , Burkardt D , Weaver DD , Gibson WT . PRC2‐complex related dysfunction in overgrowth syndromes: a review of *EZH2*, *EED*, and *SUZ12* and their syndromic phenotypes. Am J Med Genet C Semin Med Genet. 2019;181(4):519‐531. doi:10.1002/ajmg.c.31754 31724824

[13] Cao R , Wang L , Wang H , et al. Role of histone H3 lysine 27 methylation in Polycomb‐group silencing. Science. 2002;298(5595):1039‐1043. doi:10.1126/science.1076997 12351676

[14] Kuzmichev A , Nishioka K , Erdjument‐Bromage H , Tempst P , Reinberg D . Histone methyltransferase activity associated with a human multiprotein complex containing the enhancer of Zeste protein. Genes Dev. 2002;16(22):2893‐2905. doi:10.1101/gad.1035902 12435631 PMC187479

[15] Kim KH , Roberts CWM . Targeting EZH2 in cancer. Nat Med. 2016;22(2):128‐134. doi:10.1038/nm.4036 26845405 PMC4918227

[16] Eich ML , Athar M , Ferguson JE , Varambally S . EZH2‐targeted therapies in cancer: hype or a reality. Cancer Res. 2020;80(24):5449‐5458. doi:10.1158/0008-5472.CAN-20-2147 32978169 PMC8323716

[17] Morin RD , Johnson NA , Severson TM , et al. Somatic mutations altering EZH2 (Tyr641) in follicular and diffuse large B‐cell lymphomas of germinal‐center origin. Nat Genet. 2010;42(2):181‐185. doi:10.1038/ng.518 20081860 PMC2850970

[18] McCabe MT , Ott HM , Ganji G , et al. EZH2 inhibition as a therapeutic strategy for lymphoma with EZH2‐activating mutations. Nature. 2012;492(7427):108‐112. doi:10.1038/nature11606 23051747

[19] Bödör C , Grossmann V , Popov N , et al. EZH2 mutations are frequent and represent an early event in follicular lymphoma. Blood. 2013;122(18):3165‐3168. doi:10.1182/blood-2013-04-496893 24052547 PMC3814734

[20] Varambally S , Dhanasekaran SM , Zhou M , et al. The polycomb group protein EZH2 is involved in progression of prostate cancer. Nature. 2002;419(6907):624‐629. doi:10.1038/nature01075 12374981

[21] Bachmann IM , Halvorsen OJ , Collett K , et al. EZH2 expression is associated with high proliferation rate and aggressive tumor subgroups in cutaneous melanoma and cancers of the endometrium, prostate, and breast. J Clin Oncol. 2006;24(2):268‐273. doi:10.1200/JCO.2005.01.5180 16330673

[22] Wilson BG , Roberts CWM . SWI/SNF nucleosome remodellers and cancer. Nat Rev Cancer. 2011;11(7):481‐492. doi:10.1038/nrc3068 21654818

[23] Wilson BG , Wang X , Shen X , et al. Epigenetic antagonism between Polycomb and SWI/SNF complexes during oncogenic transformation. Cancer Cell. 2010;18(4):316‐328. doi:10.1016/j.ccr.2010.09.006 20951942 PMC2957473

[24] Knutson SK , Warholic NM , Wigle TJ , et al. Durable tumor regression in genetically altered malignant rhabdoid tumors by inhibition of methyltransferase EZH2. Proc Natl Acad Sci. 2013;110(19):7922‐7927. doi:10.1073/pnas.1303800110 23620515 PMC3651445

[25] Marquez VE . 3‐deazaneplanocin a (DZNep): a drug that deserves a second look. J Med Chem. 2024;67(20):17964‐17979. doi:10.1021/acs.jmedchem.4c01566 39392180

[26] Italiano A , Soria JC , Toulmonde M , et al. Tazemetostat, an EZH2 inhibitor, in relapsed or refractory B‐cell non‐Hodgkin lymphoma and advanced solid tumours: a first‐in‐human, open‐label, phase 1 study. Lancet Oncol. 2018;19(5):649‐659. doi:10.1016/S1470-2045(18)30145-1 29650362

[27] Morschhauser F , Tilly H , Chaidos A , et al. Tazemetostat for patients with relapsed or refractory follicular lymphoma: an open‐label, single‐arm, multicentre, phase 2 trial. Lancet Oncol. 2020;21(11):1433‐1442. doi:10.1016/S1470-2045(20)30441-1 33035457 PMC8427481

[28] Zhang HT , Zeng LF , He QY , Tao WA , Zha ZG , Hu CD . The E3 ubiquitin ligase CHIP mediates ubiquitination and proteasomal degradation of PRMT5. Biochim Biophys Acta Mol Cell Res. 2016;1863(2):335‐346. doi:10.1016/j.bbamcr.2015.12.001 PMC5397900 26658161

[29] Izutsu K , Ando K , Nishikori M , et al. Phase II study of tazemetostat for relapsed or refractory B‐cell non‐Hodgkin lymphoma with *EZH2* mutation in Japan. Cancer Sci. 2021;112(9):3627‐3635. doi:10.1111/cas.15040 34159682 PMC8409398

[30] Proudman DG , Gupta D , Nellesen D , et al. Tazemetostat in relapsed/refractory follicular lymphoma: a propensity score‐matched analysis of E7438‐G000‐101 trial outcomes. Oncotarget. 2022;13(1):677‐683. doi:10.18632/oncotarget.28229 35574216 PMC9093865

[31] LaFave LM , Béguelin W , Koche R , et al. Loss of BAP1 function leads to EZH2‐dependent transformation. Nat Med. 2015;21(11):1344‐1349. doi:10.1038/nm.3947 26437366 PMC4636469

[32] Zauderer MG , Szlosarek PW , Le Moulec S , et al. EZH2 inhibitor tazemetostat in patients with relapsed or refractory, BAP1‐inactivated malignant pleural mesothelioma: a multicentre, open‐label, phase 2 study. Lancet Oncol. 2022;23(6):758‐767. doi:10.1016/S1470-2045(22)00277-7 35588752

[33] Palomba ML , Cartron G , Popplewell L , et al. Combination of Atezolizumab and Tazemetostat in patients with relapsed/refractory diffuse large B‐cell lymphoma: results from a phase Ib study. Clin Lymphoma Myeloma Leuk. 2022;22(7):504‐512. doi:10.1016/j.clml.2021.12.014 35151584

[34] Sarkozy C , Morschhauser F , Dubois S , et al. A LYSA phase Ib study of Tazemetostat (EPZ‐6438) plus R‐CHOP in patients with newly diagnosed diffuse large B‐cell lymphoma (DLBCL) with poor prognosis features. Clin Cancer Res. 2020;26(13):3145‐3153. doi:10.1158/1078-0432.CCR-19-3741 32122924

[35] Yap TA , Winter JN , Giulino‐Roth L , et al. Phase I study of the novel enhancer of Zeste homolog 2 (EZH2) inhibitor GSK2816126 in patients with advanced hematologic and solid tumors. Clin Cancer Res. 2019;25(24):7331‐7339. doi:10.1158/1078-0432.CCR-18-4121 31471312 PMC7377921

[36] Frankel AE , Liu X , Minna JD . Developing EZH2‐targeted therapy for lung cancer. Cancer Discov. 2016;6(9):949‐952. doi:10.1158/2159-8290.CD-16-0800 27587466 PMC5012289

[37] Vaswani RG , Gehling VS , Dakin LA , et al. Identification of (R)‐N‐([4‐Methoxy‐6‐methyl‐2‐oxo‐1,2‐dihydropyridin‐3‐yl]methyl)‐2‐methyl‐1‐(1‐(1‐[2,2,2‐trifluoroethyl]piperidin‐4‐yl)ethyl)‐1H‐indole‐3‐carboxamide (CPI‐1205), a potent and selective inhibitor of histone methyltransferase EZH2, suitable for phase I clinical trials for B‐cell lymphomas. J Med Chem. 2016;59(21):9928‐9941. doi:10.1021/acs.jmedchem.6b01315 27739677 PMC5451150

[38] Song Y , Liu Y , Li ZM , et al. SHR2554, an EZH2 inhibitor, in relapsed or refractory mature lymphoid neoplasms: a first‐in‐human, dose‐escalation, dose‐expansion, and clinical expansion phase 1 trial. Lancet Haematol. 2022;9(7):e493‐e503. doi:10.1016/S2352-3026(22)00134-X 35772429

[39] Tachibana M , Matsuki S , Toyama K , et al. Safety, tolerability, and pharmacokinetics of Valemetostat tablets and the effect of food on Valemetostat pharmacokinetics in healthy subjects: two phase 1 studies. Clin Pharmacol Drug Dev. 2024;13(1):77‐86. doi:10.1002/cpdd.1315 37565616

[40] Izutsu K , Makita S , Nosaka K , et al. An open‐label, single‐arm phase 2 trial of valemetostat for relapsed or refractory adult T‐cell leukemia/lymphoma. Blood. 2023;141(10):1159‐1168. doi:10.1182/blood.2022016862 36150143 PMC10651775

[41] Choudhury NJ , Lai WV , Makhnin A , et al. A phase I/II study of Valemetostat (DS‐3201b), an EZH1/2 inhibitor, in combination with irinotecan in patients with recurrent small‐cell lung cancer. Clin Cancer Res. 2024;30(17):3697‐3703. doi:10.1158/1078-0432.CCR-23-3383 38940666 PMC11371507

[42] Huang Y , Sendzik M , Zhang J , et al. Discovery of the clinical candidate MAK683: An EED‐directed, allosteric, and selective PRC2 inhibitor for the treatment of advanced malignancies. J Med Chem. 2022;65(7):5317‐5333. doi:10.1021/acs.jmedchem.1c02148 35352560

[43] Krivtsov AV , Hoshii T , Armstrong SA . Mixed‐lineage leukemia fusions and chromatin in leukemia. Cold Spring Harb Perspect Med. 2017;7(11):a026658. doi:10.1101/cshperspect.a026658 28242784 PMC5666623

[44] Li Y , Han J , Zhang Y , et al. Structural basis for activity regulation of MLL family methyltransferases. Nature. 2016;530(7591):447‐452. doi:10.1038/nature16952 26886794 PMC5125619

[45] Wang P , Lin C , Smith ER , et al. Global analysis of H3K4 methylation defines MLL family member targets and points to a role for MLL1‐mediated H3K4 methylation in the regulation of transcriptional initiation by RNA polymerase II. Mol Cell Biol. 2009;29(22):6074‐6085. doi:10.1128/MCB.00924-09 19703992 PMC2772563

[46] Krivtsov AV , Armstrong SA . MLL translocations, histone modifications and leukaemia stem‐cell development. Nat Rev Cancer. 2007;7(11):823‐833. doi:10.1038/nrc2253 17957188

[47] Krivtsov AV , Twomey D , Feng Z , et al. Transformation from committed progenitor to leukaemia stem cell initiated by MLL–AF9. Nature. 2006;442(7104):818‐822. doi:10.1038/nature04980 16862118

[48] Yokoyama A , Wang Z , Wysocka J , et al. Leukemia proto‐oncoprotein MLL forms a SET1‐like histone methyltransferase complex with Menin to regulate *Hox* gene expression. Mol Cell Biol. 2004;24(13):5639‐5649. doi:10.1128/MCB.24.13.5639-5649.2004 15199122 PMC480881

[49] Milne TA , Briggs SD , Brock HW , et al. MLL targets SET domain methyltransferase activity to Hox gene promoters. Mol Cell. 2002;10(5):1107‐1117. doi:10.1016/S1097-2765(02)00741-4 12453418

[50] Kühn MWM , Song E , Feng Z , et al. Targeting chromatin regulators inhibits leukemogenic gene expression in *NPM1* mutant leukemia. Cancer Discov. 2016;6(10):1166‐1181. doi:10.1158/2159-8290.CD-16-0237 27535106 PMC5584808

[51] Döhner H , Wei AH , Appelbaum FR , et al. Diagnosis and management of AML in adults: 2022 recommendations from an international expert panel on behalf of the ELN. Blood. 2022;140(12):1345‐1377. doi:10.1182/blood.2022016867 35797463

[52] Uckelmann HJ , Kim SM , Wong EM , et al. Therapeutic targeting of preleukemia cells in a mouse model of *NPM1* mutant acute myeloid leukemia. Science. 2020;367(6477):586‐590. doi:10.1126/science.aax5863 32001657 PMC7754791

[53] Klossowski S , Miao H , Kempinska K , et al. Menin inhibitor MI‐3454 induces remission in MLL1‐rearranged and NPM1‐mutated models of leukemia. J Clin Invest. 2020;130(2):981‐997. doi:10.1172/JCI129126 31855575 PMC6994154

[54] Krivtsov AV , Evans K , Gadrey JY , et al. A Menin‐MLL inhibitor induces specific chromatin changes and eradicates disease in models of MLL‐rearranged leukemia. Cancer Cell. 2019;36(6):660‐673. doi:10.1016/j.ccell.2019.11.001 31821784 PMC7227117

[55] Borkin D , He S , Miao H , et al. Pharmacologic inhibition of the Menin‐MLL interaction blocks progression of MLL leukemia in vivo. Cancer Cell. 2015;27(4):589‐602. doi:10.1016/j.ccell.2015.02.016 25817203 PMC4415852

[56] Grembecka J , He S , Shi A , et al. Menin‐MLL inhibitors reverse oncogenic activity of MLL fusion proteins in leukemia. Nat Chem Biol. 2012;8(3):277‐284. doi:10.1038/nchembio.773 22286128 PMC3401603

[57] Yokoyama A , Somervaille TCP , Smith KS , Rozenblatt‐Rosen O , Meyerson M , Cleary ML . The Menin tumor suppressor protein is an essential oncogenic cofactor for MLL‐associated leukemogenesis. Cell. 2005;123(2):207‐218. doi:10.1016/j.cell.2005.09.025 16239140

[58] Kühn MWM , Armstrong SA . Designed to kill: novel Menin‐MLL inhibitors target MLL ‐rearranged leukemia. Cancer Cell. 2015;27(4):431‐433. doi:10.1016/j.ccell.2015.03.012 25873167

[59] Uckelmann HJ , Haarer EL , Takeda R , et al. Mutant NPM1 directly regulates oncogenic transcription in acute myeloid leukemia. Cancer Discov. 2023;13(3):746‐765. doi:10.1158/2159-8290.CD-22-0366 36455613 PMC10084473

[60] Heikamp EB , Henrich JA , Perner F , et al. The menin‐MLL1 interaction is a molecular dependency in *NUP98* ‐rearranged AML. Blood. 2022;139(6):894‐906. doi:10.1182/blood.2021012806 34582559 PMC8832476

[61] Soto‐Feliciano YM , Sánchez‐Rivera FJ , Perner F , et al. A molecular switch between mammalian MLL complexes dictates response to Menin–MLL inhibition. Cancer Discov. 2023;13(1):146‐169. doi:10.1158/2159-8290.CD-22-0416 36264143 PMC9827117

[62] Perner F , Stein EM , Wenge DV , et al. MEN1 mutations mediate clinical resistance to menin inhibition. Nature. 2023;615(7954):913‐919. doi:10.1038/s41586-023-05755-9 36922589 PMC10157896

[63] Olsen SN , Godfrey L , Healy JP , et al. MLL::AF9 degradation induces rapid changes in transcriptional elongation and subsequent loss of an active chromatin landscape. Mol Cell. 2022;82(6):1140‐1155.e11. doi:10.1016/j.molcel.2022.02.013 35245435 PMC9044330

[64] Kwon MC , Thuring JW , Querolle O , et al. Preclinical efficacy of the potent, selective menin‐KMT2A inhibitor JNJ‐75276617 (bleximenib) in *KMT2A*‐ and *NPM1*‐altered leukemias. Blood. 2024;144(11):1206‐1220. doi:10.1182/blood.2023022480 38905635 PMC11419783

[65] Issa GC , Aldoss I , DiPersio J , et al. The menin inhibitor revumenib in KMT2A‐rearranged or NPM1‐mutant leukaemia. Nature. 2023;615(7954):920‐924. doi:10.1038/s41586-023-05812-3 36922593 PMC10060155

[66] Nguyen AT , Zhang Y . The diverse functions of Dot1 and H3K79 methylation. Genes Dev. 2011;25(13):1345‐1358. doi:10.1101/gad.2057811 21724828 PMC3134078

[67] Steger DJ , Lefterova MI , Ying L , et al. DOT1L/KMT4 recruitment and H3K79 methylation are ubiquitously coupled with gene transcription in mammalian cells. Mol Cell Biol. 2008;28(8):2825‐2839. doi:10.1128/MCB.02076-07 18285465 PMC2293113

[68] Okada Y , Feng Q , Lin Y , et al. hDOT1L links histone methylation to leukemogenesis. Cell. 2005;121(2):167‐178. doi:10.1016/j.cell.2005.02.020 15851025

[69] Nguyen AT , He J , Taranova O , Zhang Y . Essential role of DOT1L in maintaining normal adult hematopoiesis. Cell Res. 2011;21(9):1370‐1373. doi:10.1038/cr.2011.115 21769133 PMC3166961

[70] Deshpande AJ , Chen L , Fazio M , et al. Leukemic transformation by the MLL‐AF6 fusion oncogene requires the H3K79 methyltransferase Dot1l. Blood. 2013;121(13):2533‐2541. doi:10.1182/blood-2012-11-465120 23361907 PMC3612861

[71] Cutler JA , Perner F , Armstrong SA . Histone PTM crosstalk stimulates Dot1 methyltransferase activity. Trends Biochem Sci. 2021;46(7):522‐524. doi:10.1016/j.tibs.2021.04.001 33879367

[72] Godfrey L . *The Role of DOT1L in MLL‐AF4 Leukaemia*, University of Oxford. 2018 http://purl.org/dc/dcmitype/Text, https://www.ora.ox.ac.uk/objects/uuid:9a1ad020-202a-4413-b9be-cdef28b2d124

[73] Okuda H , Stanojevic B , Kanai A , et al. Cooperative gene activation by AF4 and DOT1L drives MLL‐rearranged leukemia. J Clin Invest. 2017;127(5):1918‐1931. doi:10.1172/JCI91406 28394257 PMC5409830

[74] Chen CW , Armstrong SA . Targeting DOT1L and HOX gene expression in MLL‐rearranged leukemia and beyond. Exp Hematol. 2015;43(8):673‐684. doi:10.1016/j.exphem.2015.05.012 26118503 PMC4540610

[75] Bernt KM , Zhu N , Sinha AU , et al. MLL‐rearranged leukemia is dependent on aberrant H3K79 methylation by DOT1L. Cancer Cell. 2011;20(1):66‐78. doi:10.1016/j.ccr.2011.06.010 21741597 PMC3329803

[76] Krivtsov AV , Feng Z , Lemieux ME , et al. H3K79 methylation profiles define murine and human MLL‐AF4 leukemias. Cancer Cell. 2008;14(5):355‐368. doi:10.1016/j.ccr.2008.10.001 18977325 PMC2591932

[77] Kuhn MWM , Hadler MJ , Daigle SR , et al. MLL partial tandem duplication leukemia cells are sensitive to small molecule DOT1L inhibition. Haematologica. 2015;100(5):e190‐e193. doi:10.3324/haematol.2014.115337 25596271 PMC4420229

[78] Daigle SR , Olhava EJ , Therkelsen CA , et al. Selective killing of mixed lineage leukemia cells by a potent small‐molecule DOT1L inhibitor. Cancer Cell. 2011;20(1):53‐65. doi:10.1016/j.ccr.2011.06.009 21741596 PMC4046888

[79] Daigle SR , Olhava EJ , Therkelsen CA , et al. Potent inhibition of DOT1L as treatment of MLL‐fusion leukemia. Blood. 2013;122(6):1017‐1025. doi:10.1182/blood-2013-04-497644 23801631 PMC3739029

[80] Stein EM , Garcia‐Manero G , Rizzieri DA , et al. The DOT1L inhibitor pinometostat reduces H3K79 methylation and has modest clinical activity in adult acute leukemia. Blood. 2018;131(24):2661‐2669. doi:10.1182/blood-2017-12-818948 29724899 PMC6265654

[81] Perner F , Gadrey JY , Xiong Y , et al. Novel inhibitors of the histone methyltransferase DOT1L show potent antileukemic activity in patient‐derived xenografts. Blood. 2020;136(17):1983‐1988. doi:10.1182/blood.2020006113 32575123 PMC8209563

[82] Stauffer F , Weiss A , Scheufler C , et al. New potent DOT1L inhibitors for *in vivo* evaluation in mouse. ACS Med Chem Lett. 2019;10(12):1655‐1660. doi:10.1021/acsmedchemlett.9b00452 31857842 PMC6912874

[83] Kim H , Ronai ZA . PRMT5 function and targeting in cancer. Cell Stress. 2020;4(8):199‐215. doi:10.15698/cst2020.08.228 32743345 PMC7380451

[84] Antonysamy S , Bonday Z , Campbell RM , et al. Crystal structure of the human PRMT5:MEP50 complex. Proc Natl Acad Sci. 2012;109(44):17960‐17965. doi:10.1073/pnas.1209814109 23071334 PMC3497828

[85] Burgos ES , Wilczek C , Onikubo T , et al. Histone H2A and H4 N‐terminal tails are positioned by the MEP50 WD repeat protein for efficient methylation by the PRMT5 arginine methyltransferase. J Biol Chem. 2015;290(15):9674‐9689. doi:10.1074/jbc.M115.636894 25713080 PMC4392268

[86] Migliori V , Müller J , Phalke S , et al. Symmetric dimethylation of H3R2 is a newly identified histone mark that supports euchromatin maintenance. Nat Struct Mol Biol. 2012;19(2):136‐144. doi:10.1038/nsmb.2209 22231400

[87] Pal S , Baiocchi RA , Byrd JC , Grever MR , Jacob ST , Sif S . Low levels of miR‐92b/96 induce PRMT5 translation and H3R8/H4R3 methylation in mantle cell lymphoma. EMBO J. 2007;26(15):3558‐3569. doi:10.1038/sj.emboj.7601794 17627275 PMC1949000

[88] Wang L , Pal S , Sif S . Protein arginine methyltransferase 5 suppresses the transcription of the RB family of tumor suppressors in leukemia and lymphoma cells. Mol Cell Biol. 2008;28(20):6262‐6277. doi:10.1128/MCB.00923-08 18694959 PMC2577430

[89] Nie M , Wang Y , Guo C , et al. CARM1‐mediated methylation of protein arginine methyltransferase 5 represses human γ‐globin gene expression in erythroleukemia cells. J Biol Chem. 2018;293(45):17454‐17463. doi:10.1074/jbc.RA118.004028 30257864 PMC6231142

[90] Espejo AB , Gao G , Black K , et al. PRMT5 C‐terminal phosphorylation modulates a 14‐3‐3/PDZ interaction switch. J Biol Chem. 2017;292(6):2255‐2265. doi:10.1074/jbc.M116.760330 28031468 PMC5313098

[91] Lattouf H , Poulard C , Le Romancer M . PRMT5 prognostic value in cancer. Oncotarget. 2019;10(34):3151‐3153. doi:10.18632/oncotarget.26883 31139329 PMC6516714

[92] Tarighat SS , Santhanam R , Frankhouser D , et al. The dual epigenetic role of PRMT5 in acute myeloid leukemia: gene activation and repression via histone arginine methylation. Leukemia. 2016;30(4):789‐799. doi:10.1038/leu.2015.308 26536822 PMC8034866

[93] Radzisheuskaya A , Shliaha PV , Grinev V , et al. PRMT5 methylome profiling uncovers a direct link to splicing regulation in acute myeloid leukemia. Nat Struct Mol Biol. 2019;26(11):999‐1012. doi:10.1038/s41594-019-0313-z 31611688 PMC6858565

[94] Huang X , Yang Y , Zhu D , et al. PRMT5‐mediated RNF4 methylation promotes therapeutic resistance of APL cells to As2O3 by stabilizing oncoprotein PML‐RARα. Cell Mol Life Sci. 2022;79(6):319. doi:10.1007/s00018-022-04358-3 35622143 PMC11072021

[95] Liu F , Zhao X , Perna F , et al. JAK2V617F‐mediated phosphorylation of PRMT5 downregulates its methyltransferase activity and promotes myeloproliferation. Cancer Cell. 2011;19(2):283‐294. doi:10.1016/j.ccr.2010.12.020 21316606 PMC4687747

[96] Jin Y , Zhou J , Xu F , et al. Targeting methyltransferase PRMT5 eliminates leukemia stem cells in chronic myelogenous leukemia. J Clin Invest. 2016;126(10):3961‐3980. doi:10.1172/JCI85239 27643437 PMC5096815

[97] Curry E , Green I , Chapman‐Rothe N , et al. Dual EZH2 and EHMT2 histone methyltransferase inhibition increases biological efficacy in breast cancer cells. Clin Epigenetics. 2015;7(1):84. doi:10.1186/s13148-015-0118-9 26300989 PMC4545913

[98] Sbirkov Y , Schenk T , Kwok C , et al. Dual inhibition of EZH2 and G9A/GLP histone methyltransferases by HKMTI‐1‐005 promotes differentiation of acute myeloid leukemia cells. Front Cell Dev Biol. 2023;11:1076458. doi:10.3389/fcell.2023.1076458 37035245 PMC10076884

[99] Spiliopoulou P , Spear S , Mirza H , et al. Dual G9A/EZH2 inhibition stimulates antitumor immune response in ovarian high‐grade serous carcinoma. Mol Cancer Ther. 2022;21(4):522‐534. doi:10.1158/1535-7163.MCT-21-0743 35131874 PMC9377747

[100] Shi Y , Lan F , Matson C , et al. Histone demethylation mediated by the nuclear amine oxidase homolog LSD1. Cell. 2004;119(7):941‐953. doi:10.1016/j.cell.2004.12.012 15620353

[101] Hakimi MA , Bochar DA , Chenoweth J , Lane WS , Mandel G , Shiekhattar R . A core‐BRAF35 complex containing histone deacetylase mediates repression of neuronal‐specific genes. Proc Natl Acad Sci U S A. 2002;99(11):7420‐7425. doi:10.1073/pnas.112008599 12032298 PMC124246

[102] Hakimi MA , Dong Y , Lane WS , Speicher DW , Shiekhattar R . A candidate X‐linked mental retardation gene is a component of a new family of histone deacetylase‐containing complexes. J Biol Chem. 2003;278(9):7234‐7239. doi:10.1074/jbc.M208992200 12493763

[103] Shi Y , Sawada J i , Sui G , et al. Coordinated histone modifications mediated by a CtBP co‐repressor complex. Nature. 2003;422(6933):735‐738. doi:10.1038/nature01550 12700765

[104] Tong JK , Hassig CA , Schnitzler GR , Kingston RE , Schreiber SL . Chromatin deacetylation by an ATP‐dependent nucleosome remodelling complex. Nature. 1998;395(6705):917‐921. doi:10.1038/27699 9804427

[105] You A , Tong JK , Grozinger CM , Schreiber SL . CoREST is an integral component of the CoREST‐ human histone deacetylase complex. Proc Natl Acad Sci U S A. 2001;98(4):1454‐1458. doi:10.1073/pnas.98.4.1454 11171972 PMC29278

[106] Humphrey GW , Wang Y , Russanova VR , et al. Stable histone deacetylase complexes distinguished by the presence of SANT domain proteins CoREST/kiaa0071 and Mta‐L1. J Biol Chem. 2001;276(9):6817‐6824. doi:10.1074/jbc.M007372200 11102443

[107] Metzger E , Wissmann M , Yin N , et al. LSD1 demethylates repressive histone marks to promote androgen‐receptor‐dependent transcription. Nature. 2005;437(7057):436‐439. doi:10.1038/nature04020 16079795

[108] Mendenhall EM , Williamson KE , Reyon D , et al. Locus‐specific editing of histone modifications at endogenous enhancers. Nat Biotechnol. 2013;31(12):1133‐1136. doi:10.1038/nbt.2701 24013198 PMC3858395

[109] Kearns NA , Pham H , Tabak B , et al. Functional annotation of native enhancers with a Cas9‐histone demethylase fusion. Nat Methods. 2015;12(5):401‐403. doi:10.1038/nmeth.3325 25775043 PMC4414811

[110] Saleque S , Kim J , Rooke HM , Orkin SH . Epigenetic regulation of hematopoietic differentiation by Gfi‐1 and Gfi‐1b is mediated by the cofactors CoREST and LSD1. Mol Cell. 2007;27(4):562‐572. doi:10.1016/j.molcel.2007.06.039 17707228

[111] Maiques‐Diaz A , Somervaille TC . LSD1: biologic roles and therapeutic targeting. Epigenomics. 2016;8(8):1103‐1116. doi:10.2217/epi-2016-0009 27479862 PMC5066116

[112] Wang J , Hevi S , Kurash JK , et al. The lysine demethylase LSD1 (KDM1) is required for maintenance of global DNA methylation. Nat Genet. 2009;41(1):125‐129. doi:10.1038/ng.268 19098913

[113] Wang J , Scully K , Zhu X , et al. Opposing LSD1 complexes function in developmental gene activation and repression programmes. Nature. 2007;446(7138):882‐887. doi:10.1038/nature05671 17392792

[114] Kerenyi MA , Shao Z , Hsu YJ , et al. Histone demethylase Lsd1 represses hematopoietic stem and progenitor cell signatures during blood cell maturation. eLife. 2013;2:e00633. doi:10.7554/eLife.00633 23795291 PMC3687337

[115] Sprüssel A , Schulte JH , Weber S , et al. Lysine‐specific demethylase 1 restricts hematopoietic progenitor proliferation and is essential for terminal differentiation. Leukemia. 2012;26(9):2039‐2051. doi:10.1038/leu.2012.157 22699452

[116] Duteil D , Metzger E , Willmann D , et al. LSD1 promotes oxidative metabolism of white adipose tissue. Nat Commun. 2014;5(1):4093. doi:10.1038/ncomms5093 24912735 PMC4112219

[117] Duteil D , Tosic M , Lausecker F , et al. Lsd1 ablation triggers metabolic reprogramming of Brown adipose tissue. Cell Rep. 2016;17(4):1008‐1021. doi:10.1016/j.celrep.2016.09.053 27760309 PMC5081406

[118] Suzuki J , Maruyama S , Tamauchi H , et al. Gfi1, a transcriptional repressor, inhibits the induction of the T helper type 1 programme in activated CD4 T cells. Immunology. 2016;147(4):476‐487. doi:10.1111/imm.12580 26749286 PMC4799889

[119] Sun G , Alzayady K , Stewart R , et al. Histone demethylase LSD1 regulates neural stem cell proliferation. Mol Cell Biol. 2010;30(8):1997‐2005. doi:10.1128/MCB.01116-09 20123967 PMC2849468

[120] Subramaniam A , Žemaitis K , Talkhoncheh MS , et al. Lysine‐specific demethylase 1A restricts ex vivo propagation of human HSCs and is a target of UM171. Blood. 2020;136(19):2151‐2161. doi:10.1182/blood.2020005827 32582923 PMC7645986

[121] Chagraoui J , Girard S , Spinella JF , et al. UM171 preserves epigenetic Marks that are reduced in ex vivo culture of human HSCs via potentiation of the CLR3‐KBTBD4 complex. Cell Stem Cell. 2021;28(1):48‐62.e6. doi:10.1016/j.stem.2020.12.002 33417871

[122] Etani T , Naiki T , Naiki‐Ito A , et al. NCL1, a highly selective lysine‐specific demethylase 1 inhibitor, suppresses castration‐resistant prostate cancer growth via regulation of apoptosis and autophagy. J Clin Med. 2019;8(4):442. doi:10.3390/jcm8040442 30935141 PMC6517972

[123] Zhou M , Venkata PP , Viswanadhapalli S , et al. KDM1A inhibition is effective in reducing stemness and treating triple negative breast cancer. Breast Cancer Res Treat. 2021;185(2):343‐357. doi:10.1007/s10549-020-05963-1 33057995

[124] Jin Y , Ma D , Gramyk T , et al. Kdm1a promotes SCLC progression by transcriptionally silencing the tumor suppressor Rest. Biochem Biophys Res Commun. 2019;515(1):214‐221. doi:10.1016/j.bbrc.2019.05.118 31146914

[125] Lee C , Rudneva VA , Erkek S , et al. Lsd1 as a therapeutic target in Gfi1‐activated medulloblastoma. Nat Commun. 2019;10(1):332. doi:10.1038/s41467-018-08269-5 30659187 PMC6338772

[126] Zhang S , Liu M , Yao Y , Yu B , Liu H . Targeting LSD1 for acute myeloid leukemia (AML) treatment. Pharmacol Res. 2021;164:105335. doi:10.1016/j.phrs.2020.105335 33285227

[127] Noce B , Di Bello E , Fioravanti R , Mai A . LSD1 inhibitors for cancer treatment: focus on multi‐target agents and compounds in clinical trials. Front Pharmacol. 2023;14. doi:10.3389/fphar.2023.1120911 PMC9932783 36817147

[128] Harris WJ , Huang X , Lynch JT , et al. The histone demethylase KDM1A sustains the oncogenic potential of MLL‐AF9 leukemia stem cells. Cancer Cell. 2012;21(4):473‐487. doi:10.1016/j.ccr.2012.03.014 22464800

[129] Schenk T , Chen WC , Göllner S , et al. Inhibition of the LSD1 (KDM1A) demethylase reactivates the all‐trans‐retinoic acid differentiation pathway in acute myeloid leukemia. Nat Med. 2012;18(4):605‐611. doi:10.1038/nm.2661 22406747 PMC3539284

[130] Maiques‐Diaz A , Spencer GJ , Lynch JT , et al. Enhancer activation by pharmacologic displacement of LSD1 from GFI1 induces differentiation in acute myeloid leukemia. Cell Rep. 2018;22(13):3641‐3659. doi:10.1016/j.celrep.2018.03.012 29590629 PMC5896174

[131] Barth J , Abou‐El‐Ardat K , Dalic D , et al. LSD1 inhibition by tranylcypromine derivatives interferes with GFI1‐mediated repression of PU.1 target genes and induces differentiation in AML. Leukemia. 2019;33(6):1411‐1426. doi:10.1038/s41375-018-0375-7 30679800

[132] Hartung EE , Singh K , Berg T . LSD1 inhibition modulates transcription factor networks in myeloid malignancies. Front Oncol. 2023;13:1149754. doi:10.3389/fonc.2023.1149754 36969082 PMC10036816

[133] Cusan M , Cai SF , Mohammad HP , et al. LSD1 inhibition exerts its antileukemic effect by recommissioning PU.1‐ and C/EBPα‐dependent enhancers in AML. Blood. 2018;131(15):1730‐1742. doi:10.1182/blood-2017-09-807024 29453291 PMC5897868

[134] Bell CC , Fennell KA , Chan YC , et al. Targeting enhancer switching overcomes non‐genetic drug resistance in acute myeloid leukaemia. Nat Commun. 2019;10(1):2723. doi:10.1038/s41467-019-10652-9 31222014 PMC6586637

[135] Cai SF , Chu SH , Goldberg AD , et al. Leukemia cell of origin influences apoptotic priming and sensitivity to LSD1 inhibition. Cancer Discov. 2020;10(10):1500‐1513. doi:10.1158/2159-8290.CD-19-1469 32606137 PMC7584353

[136] Jutzi JS , Kleppe M , Dias J , et al. LSD1 inhibition prolongs survival in mouse models of MPN by selectively targeting the disease clone. HemaSphere. 2018;2(3):e54. doi:10.1097/HS9.0000000000000054 31723778 PMC6745991

[137] Mohammad HP , Smitheman KN , Kamat CD , et al. A DNA hypomethylation signature predicts antitumor activity of LSD1 inhibitors in SCLC. Cancer Cell. 2015;28(1):57‐69. doi:10.1016/j.ccell.2015.06.002 26175415

[138] Mohammad HP , Kruger RG . Antitumor activity of LSD1 inhibitors in lung cancer. Mol Cell Oncol. 2016;3(2):e1117700. doi:10.1080/23723556.2015.1117700 27308632 PMC4905412

[139] Takagi S , Ishikawa Y , Mizutani A , et al. LSD1 inhibitor T‐3775440 inhibits SCLC cell proliferation by disrupting LSD1 interactions with SNAG domain proteins INSM1 and GFI1B. Cancer Res. 2017;77(17):4652‐4662. doi:10.1158/0008-5472.CAN-16-3502 28667074

[140] Augert A , Eastwood E , Ibrahim AH , et al. Targeting NOTCH activation in small cell lung cancer through LSD1 inhibition. Sci Signal. 2019;12(567):eaau2922. doi:10.1126/scisignal.aau2922 30723171 PMC6530478

[141] Hiatt JB , Sandborg H , Garrison SM , et al. Inhibition of LSD1 with Bomedemstat sensitizes small cell lung cancer to immune checkpoint blockade and T‐cell killing. Clin Cancer Res. 2022;28(20):4551‐4564. doi:10.1158/1078-0432.CCR-22-1128 35920742 PMC9844673

[142] Nguyen EM , Taniguchi H , Chan JM , et al. Targeting lysine‐specific demethylase 1 rescues major histocompatibility complex class I antigen presentation and overcomes programmed death‐ligand 1 blockade resistance in SCLC. J Thorac Oncol. 2022;17(8):1014‐1031. doi:10.1016/j.jtho.2022.05.014 35691495 PMC9357096

[143] Chen K , Cai Y , Cheng C , et al. MYT1 attenuates neuroblastoma cell differentiation by interacting with the LSD1/CoREST complex. Oncogene. 2020;39(21):4212‐4226. doi:10.1038/s41388-020-1268-6 32251364

[144] Schulte JH , Lim S , Schramm A , et al. Lysine‐specific demethylase 1 is strongly expressed in poorly differentiated neuroblastoma: implications for therapy. Cancer Res. 2009;69(5):2065‐2071. doi:10.1158/0008-5472.CAN-08-1735 19223552

[145] Li Y , Zhao Y , Li X , et al. Biological and therapeutic role of LSD1 in Alzheimer's diseases. Front Pharmacol. 2022;13:1020556. doi:10.3389/fphar.2022.1020556 PMC9640401 36386192

[146] Schmidt DMZ , McCafferty DG . Trans‐2‐Phenylcyclopropylamine is a mechanism‐based inactivator of the histone demethylase LSD1. Biochemistry. 2007;46(14):4408‐4416. doi:10.1021/bi0618621 17367163

[147] Wass M , Göllner S , Besenbeck B , et al. A proof of concept phase I/II pilot trial of LSD1 inhibition by tranylcypromine combined with ATRA in refractory/relapsed AML patients not eligible for intensive therapy. Leukemia. 2021;35(3):701‐711. doi:10.1038/s41375-020-0892-z 32561840 PMC7303943

[148] Tayari MM , Santos HGD , Kwon D , et al. Clinical responsiveness to all‐trans retinoic acid is potentiated by LSD1 inhibition and associated with a quiescent transcriptome in myeloid malignancies. Clin Cancer Res. 2021;27(7):1893‐1903. doi:10.1158/1078-0432.CCR-20-4054 33495312 PMC8026558

[149] Lübbert M , Schmoor C , Berg T , et al. Phase I study of the LSD1 inhibitor tranylcypromine (TCP) in combination with all‐ trans retinoic acid (ATRA) and Low‐dose cytarabine (LDAC) in elderly, medically non‐fit patients with AML or high‐risk MDS (TRANSATRA trial). Blood. 2022;140:9087‐9088. doi:10.1182/blood-2022-159471

[150] Salamero O , Montesinos P , Willekens C , et al. First‐in‐human phase I study of Iadademstat (ORY‐1001): a first‐in‐class lysine‐specific histone demethylase 1A inhibitor, in relapsed or refractory acute myeloid leukemia. J Clin Oncol off J Am Soc Clin Oncol. 2020;38(36):4260‐4273. doi:10.1200/JCO.19.03250 PMC7768337 33052756

[151] Salamero O , Molero A , Pérez‐Simón JA , et al. Iadademstat in combination with azacitidine in patients with newly diagnosed acute myeloid leukaemia (ALICE): an open‐label, phase 2a dose‐finding study. Lancet Haematol. 2024;11(7):e487‐e498. doi:10.1016/S2352-3026(24)00132-7 38824932

[152] DiNardo CD , Jonas BA , Pullarkat V , et al. Azacitidine and Venetoclax in previously untreated acute myeloid leukemia. N Engl J Med. 2020;383(7):617‐629. doi:10.1056/NEJMoa2012971 32786187

[153] Singh K , Hartung E , Muhs C , et al. LSD1 inhibition synergizes with Venetoclax in acute myeloid leukemia by targeting cellular metabolism. Blood. 2023;142(Supplement 1):4156. doi:10.1182/blood-2023-189515

[154] Gill H . Phase 2 Study to Assess the Safety and Efficacy of Bomedemstat (MK3543) in Combination with Ruxolitinib in Patients with Myelofibrosis. In: ASH. 2023 https://ash.confex.com/ash/2023/webprogram/Paper181971.html

[155] Goethert J . Bomedemstat (IMG‐7289), an LSD1 Inhibitor, Manages the Signs and Symptoms of Essential Thrombocythemia (ET) While Reducing the Burden of Cells Homozygous for Driver Mutations. In: ASH. 2023 https://ash.confex.com/ash/2023/webprogram/Paper179329.html

[156] Bauer TM , Besse B , Martinez‐Marti A , et al. Phase I, open‐label, dose‐escalation study of the safety, pharmacokinetics, pharmacodynamics, and efficacy of GSK2879552 in relapsed/refractory SCLC. J Thorac Oncol. 2019;14(10):1828‐1838. doi:10.1016/j.jtho.2019.06.021 31260835

[157] Mendivil AFN , Gutierrez S , Bullock R , Buesa C . 1806P final safety and efficacy data from CLEPSIDRA trial in 2L ED‐SCLC. Ann Oncol. 2020;31:S1044. doi:10.1016/j.annonc.2020.08.1567

[158] Antonijoan RM , Ferrero‐Cafiero JM , Coimbra J , et al. First‐in‐human randomized trial to assess safety, tolerability, pharmacokinetics and pharmacodynamics of the KDM1A inhibitor Vafidemstat. CNS Drugs. 2021;35(3):331‐344. doi:10.1007/s40263-021-00797-x 33755924 PMC7985749

[159] Struys EA , Salomons GS , Achouri Y , et al. Mutations in the d‐2‐hydroxyglutarate dehydrogenase gene cause d‐2‐Hydroxyglutaric aciduria. Am J Hum Genet. 2005;76(2):358‐360. doi:10.1086/427890 15609246 PMC1196381

[160] Yan H , Parsons DW , Jin G , et al. IDH1 and IDH2 mutations in gliomas. N Engl J Med. 2009;360(8):765‐773. doi:10.1056/NEJMoa0808710 19228619 PMC2820383

[161] Mardis ER , Ding L , Dooling DJ , et al. Recurring mutations found by sequencing an acute myeloid leukemia genome. N Engl J Med. 2009;361(11):1058‐1066. doi:10.1056/NEJMoa0903840 19657110 PMC3201812

[162] Dang L , White DW , Gross S , et al. Cancer‐associated IDH1 mutations produce 2‐hydroxyglutarate. Nature. 2009;462(7274):739‐744. doi:10.1038/nature08617 19935646 PMC2818760

[163] Xu W , Yang H , Liu Y , et al. Oncometabolite 2‐hydroxyglutarate is a competitive inhibitor of α‐ketoglutarate‐dependent dioxygenases. Cancer Cell. 2011;19(1):17‐30. doi:10.1016/j.ccr.2010.12.014 21251613 PMC3229304

[164] Gross S , Cairns RA , Minden MD , et al. Cancer‐associated metabolite 2‐hydroxyglutarate accumulates in acute myelogenous leukemia with isocitrate dehydrogenase 1 and 2 mutations. J Exp Med. 2010;207(2):339‐344. doi:10.1084/jem.20092506 20142433 PMC2822606

[165] Tefferi A , Lasho TL , Abdel‐Wahab O , et al. IDH1 and IDH2 mutation studies in 1473 patients with chronic‐, fibrotic‐ or blast‐phase essential thrombocythemia, polycythemia vera or myelofibrosis. Leukemia. 2010;24(7):1302‐1309. doi:10.1038/leu.2010.113 20508616 PMC3035975

[166] Papaemmanuil E , Gerstung M , Malcovati L , et al. Clinical and biological implications of driver mutations in myelodysplastic syndromes. Blood. 2013;122(22):3616‐3627. doi:10.1182/blood-2013-08-518886 24030381 PMC3837510

[167] Papaemmanuil E , Gerstung M , Bullinger L , et al. Genomic classification and prognosis in acute myeloid leukemia. N Engl J Med. 2016;374(23):2209‐2221. doi:10.1056/NEJMoa1516192 27276561 PMC4979995

[168] Makishima H , Yoshizato T , Yoshida K , et al. Dynamics of clonal evolution in myelodysplastic syndromes. Nat Genet. 2017;49(2):204‐212. doi:10.1038/ng.3742 27992414 PMC8210656

[169] Nobusawa S , Watanabe T , Kleihues P , Ohgaki H . IDH1 mutations as molecular signature and predictive factor of secondary glioblastomas. Clin Cancer Res. 2009;15(19):6002‐6007. doi:10.1158/1078-0432.CCR-09-0715 19755387

[170] Abbas S , Lugthart S , Kavelaars FG , et al. Acquired mutations in the genes encoding IDH1 and IDH2 both are recurrent aberrations in acute myeloid leukemia: prevalence and prognostic value. Blood. 2010;116(12):2122‐2126. doi:10.1182/blood-2009-11-250878 20538800

[171] Kosmider O , Gelsi‐Boyer V , Slama L , et al. Mutations of IDH1 and IDH2 genes in early and accelerated phases of myelodysplastic syndromes and MDS/myeloproliferative neoplasms. Leukemia. 2010;24(5):1094‐1096. doi:10.1038/leu.2010.52 20376084

[172] Thol F , Weissinger EM , Krauter J , et al. IDH1 mutations in patients with myelodysplastic syndromes are associated with an unfavorable prognosis. Haematologica. 2010;95(10):1668‐1674. doi:10.3324/haematol.2010.025494 20494930 PMC2948091

[173] Simonin M , Schmidt A , Bontoux C , et al. Oncogenetic landscape and clinical impact of IDH1 and IDH2 mutations in T‐ALL. J Hematol Oncol. 2021;14(1):74. doi:10.1186/s13045-021-01068-4 33941203 PMC8091755

[174] Jusakul A , Cutcutache I , Yong CH , et al. Whole‐genome and epigenomic landscapes of etiologically distinct subtypes of cholangiocarcinoma. Cancer Discov. 2017;7(10):1116‐1135. doi:10.1158/2159-8290.CD-17-0368 28667006 PMC5628134

[175] Amary MF , Bacsi K , Maggiani F , et al. IDH1 and IDH2 mutations are frequent events in central chondrosarcoma and central and periosteal chondromas but not in other mesenchymal tumours. J Pathol. 2011;224(3):334‐343. doi:10.1002/path.2913 21598255

[176] Green A , Beer P . Somatic mutations of IDH1 and IDH2 in the leukemic transformation of myeloproliferative neoplasms. N Engl J Med. 2010;362(4):369‐370. doi:10.1056/NEJMc0910063 20107228

[177] Grassian AR , Parker SJ , Davidson SM , et al. IDH1 mutations alter citric acid cycle metabolism and increase dependence on oxidative mitochondrial metabolism. Cancer Res. 2014;74(12):3317‐3331. doi:10.1158/0008-5472.CAN-14-0772-T 24755473 PMC4885639

[178] Chan SM , Thomas D , Corces‐Zimmerman MR , et al. Isocitrate dehydrogenase 1 and 2 mutations induce BCL‐2 dependence in acute myeloid leukemia. Nat Med. 2015;21(2):178‐184. doi:10.1038/nm.3788 25599133 PMC4406275

[179] Chesnelong C , Chaumeil MM , Blough MD , et al. Lactate dehydrogenase a silencing in IDH mutant gliomas. Neuro‐Oncol. 2014;16(5):686‐695. doi:10.1093/neuonc/not243 24366912 PMC3984548

[180] Heuser M , Araujo Cruz MM , Goparaju R , Chaturvedi A . Enigmas of IDH mutations in hematology/oncology. Exp Hematol. 2015;43(8):685‐697. doi:10.1016/j.exphem.2015.05.005 26032956

[181] Zhao S , Lin Y , Xu W , et al. Glioma‐derived mutations in IDH1 dominantly inhibit IDH1 catalytic activity and induce HIF‐1alpha. Science. 2009;324(5924):261‐265. doi:10.1126/science.1170944 19359588 PMC3251015

[182] Chaturvedi A , Araujo Cruz MM , Jyotsana N , et al. Mutant IDH1 promotes leukemogenesis in vivo and can be specifically targeted in human AML. Blood. 2013;122(16):2877‐2887. doi:10.1182/blood-2013-03-491571 23954893

[183] Lu C , Ward PS , Kapoor GS , et al. IDH mutation impairs histone demethylation and results in a block to cell differentiation. Nature. 2012;483(7390):474‐478. doi:10.1038/nature10860 22343901 PMC3478770

[184] Figueroa ME , Abdel‐Wahab O , Lu C , et al. Leukemic IDH1 and IDH2 mutations result in a hypermethylation phenotype, disrupt TET2 function, and impair hematopoietic differentiation. Cancer Cell. 2010;18(6):553‐567. doi:10.1016/j.ccr.2010.11.015 21130701 PMC4105845

[185] Kernytsky A , Wang F , Hansen E , et al. IDH2 mutation‐induced histone and DNA hypermethylation is progressively reversed by small‐molecule inhibition. Blood. 2015;125(2):296‐303. doi:10.1182/blood-2013-10-533604 25398940 PMC4295919

[186] Chowdhury R , Yeoh KK , Tian YM , et al. The oncometabolite 2‐hydroxyglutarate inhibits histone lysine demethylases. EMBO Rep. 2011;12(5):463‐469. doi:10.1038/embor.2011.43 21460794 PMC3090014

[187] Noushmehr H , Weisenberger DJ , Diefes K , et al. Identification of a CpG Island methylator phenotype that defines a distinct subgroup of glioma. Cancer Cell. 2010;17(5):510‐522. doi:10.1016/j.ccr.2010.03.017 20399149 PMC2872684

[188] Ko M , Huang Y , Jankowska AM , et al. Impaired hydroxylation of 5‐methylcytosine in myeloid cancers with mutant TET2. Nature. 2010;468(7325):839‐843. doi:10.1038/nature09586 21057493 PMC3003755

[189] Flavahan WA , Drier Y , Liau BB , et al. Insulator dysfunction and oncogene activation in IDH mutant gliomas. Nature. 2016;529(7584):110‐114. doi:10.1038/nature16490 26700815 PMC4831574

[190] Losman JA , Looper RE , Koivunen P , et al. (R)‐2‐hydroxyglutarate is sufficient to promote leukemogenesis and its effects are reversible. Science. 2013;339(6127):1621‐1625. doi:10.1126/science.1231677 23393090 PMC3836459

[191] Esmaeili M , Hamans BC , Navis AC , et al. IDH1 R132H mutation generates a distinct phospholipid metabolite profile in glioma. Cancer Res. 2014;74(17):4898‐4907. doi:10.1158/0008-5472.CAN-14-0008 25005896

[192] Ward PS , Lu C , Cross JR , et al. The potential for isocitrate dehydrogenase mutations to produce 2‐hydroxyglutarate depends on allele specificity and subcellular compartmentalization. J Biol Chem. 2013;288(6):3804‐3815. doi:10.1074/jbc.M112.435495 23264629 PMC3567635

[193] Thomas D , Wu M , Nakauchi Y , et al. Dysregulated lipid synthesis by oncogenic IDH1 mutation is a targetable synthetic lethal vulnerability. Cancer Discov. 2023;13(2):496‐515. doi:10.1158/2159-8290.CD-21-0218 36355448 PMC9900324

[194] Popovici‐Muller J , Lemieux RM , Artin E , et al. Discovery of AG‐120 (Ivosidenib): a first‐in‐class mutant IDH1 inhibitor for the treatment of IDH1 mutant cancers. ACS Med Chem Lett. 2018;9(4):300‐305. doi:10.1021/acsmedchemlett.7b00421 29670690 PMC5900343

[195] DiNardo CD , Stein EM , de Botton S , et al. Durable remissions with Ivosidenib in IDH1‐mutated relapsed or refractory AML. N Engl J Med. 2018;378(25):2386‐2398. doi:10.1056/NEJMoa1716984 29860938

[196] Roboz GJ , DiNardo CD , Stein EM , et al. Ivosidenib induces deep durable remissions in patients with newly diagnosed IDH1‐mutant acute myeloid leukemia. Blood. 2020;135(7):463‐471. doi:10.1182/blood.2019002140 31841594 PMC7019193

[197] DiNardo CD , Roboz GJ , Watts JM , et al. Final phase 1 substudy results of ivosidenib for patients with mutant IDH1 relapsed/refractory myelodysplastic syndrome. Blood Adv. 2024;8(15):4209‐4220. doi:10.1182/bloodadvances.2023012302 38640348 PMC11372395

[198] Chaturvedi A , Gupta C , Gabdoulline R , et al. Synergistic activity of IDH1 inhibitor BAY1436032 with azacitidine in IDH1 mutant acute myeloid leukemia. Haematologica. 2021;106(2):565‐573. doi:10.3324/haematol.2019.236992 32241846 PMC7849562

[199] DiNardo CD , Stein AS , Stein EM , et al. Mutant isocitrate dehydrogenase 1 inhibitor Ivosidenib in combination with Azacitidine for newly diagnosed acute myeloid leukemia. J Clin Oncol. 2021;39(1):57‐65. doi:10.1200/JCO.20.01632 33119479 PMC7771719

[200] Montesinos P , Recher C , Vives S , et al. Ivosidenib and Azacitidine in IDH1‐mutated acute myeloid leukemia. N Engl J Med. 2022;386(16):1519‐1531. doi:10.1056/NEJMoa2117344 35443108

[201] Zhu AX , Macarulla T , Javle MM , et al. Final overall survival efficacy results of Ivosidenib for patients with advanced cholangiocarcinoma with IDH1 mutation: the phase 3 randomized clinical ClarIDHy trial. JAMA Oncol. 2021;7(11):1669‐1677. doi:10.1001/jamaoncol.2021.3836 34554208 PMC8461552

[202] Yen K , Travins J , Wang F , et al. AG‐221, a first‐in‐class therapy targeting acute myeloid leukemia harboring oncogenic IDH2 mutations. Cancer Discov. 2017;7(5):478‐493. doi:10.1158/2159-8290.CD-16-1034 28193778

[203] Stein EM , DiNardo CD , Pollyea DA , et al. Enasidenib in mutant IDH2 relapsed or refractory acute myeloid leukemia. Blood. 2017;130(6):722‐731. doi:10.1182/blood-2017-04-779405 28588020 PMC5572791

[204] de Botton S , Montesinos P , Schuh AC , et al. Enasidenib vs conventional care in older patients with late‐stage mutant‐IDH2 relapsed/refractory AML: a randomized phase 3 trial. Blood. 2023;141(2):156‐167. doi:10.1182/blood.2021014901 35714312 PMC10644040

[205] Cai SF , Huang Y , Lance JR , et al. A study to assess the efficacy of enasidenib and risk‐adapted addition of azacitidine in newly diagnosed IDH2‐mutant AML. Blood Adv. 2024;8(2):429‐440. doi:10.1182/bloodadvances.2023010563 37871309 PMC10827405

[206] DiNardo CD , Schuh AC , Stein EM , et al. Enasidenib plus azacitidine versus azacitidine alone in patients with newly diagnosed, mutant‐IDH2 acute myeloid leukaemia (AG221‐AML‐005): a single‐arm, phase 1b and randomised, phase 2 trial. Lancet Oncol. 2021;22(11):1597‐1608. doi:10.1016/S1470-2045(21)00494-0 34672961

[207] Caravella JA , Lin J , Diebold RB , et al. Structure‐based design and identification of FT‐2102 (Olutasidenib), a potent mutant‐selective IDH1 inhibitor. J Med Chem. 2020;63(4):1612‐1623. doi:10.1021/acs.jmedchem.9b01423 31971798

[208] Cortes J , Jurcic J , Baer M , et al. Olutasidenib for the treatment of mIDH1 acute myeloid leukemia in patients relapsed or refractory to hematopoietic stem cell transplant, prior mIDH1 inhibitor, or Venetoclax. Blood. 2023;142:2888. doi:10.1182/blood-2023-187861

[209] Watts JM , Baer MR , Yang J , et al. Olutasidenib alone or with azacitidine in IDH1‐mutated acute myeloid leukaemia and myelodysplastic syndrome: phase 1 results of a phase 1/2 trial. Lancet Haematol. 2023;10(1):e46‐e58. doi:10.1016/S2352-3026(22)00292-7 36370742 PMC12250719

[210] de Botton S , Fenaux P , Yee K , et al. Olutasidenib (FT‐2102) induces durable complete remissions in patients with relapsed or refractory IDH1‐mutated AML. Blood Adv. 2023;7(13):3117‐3127. doi:10.1182/bloodadvances.2022009411 36724515 PMC10362540

[211] Konteatis Z , Artin E , Nicolay B , et al. Vorasidenib (AG‐881): a first‐in‐class, brain‐penetrant dual inhibitor of mutant IDH1 and 2 for treatment of glioma. ACS Med Chem Lett. 2020;11(2):101‐107. doi:10.1021/acsmedchemlett.9b00509 32071674 PMC7025383

[212] Mellinghoff IK , van den Bent MJ , Blumenthal DT , et al. Vorasidenib in IDH1‐ or IDH2‐mutant Low‐grade glioma. N Engl J Med. 2023;389(7):589‐601. doi:10.1056/NEJMoa2304194 37272516 PMC11445763

[213] Bill M , Jentzsch M , Bischof L , et al. Impact of IDH1 and IDH2 mutation detection at diagnosis and in remission in patients with AML receiving allogeneic transplantation. Blood Adv. 2023;7(3):436‐444. doi:10.1182/bloodadvances.2021005789 35381077 PMC9979713

[214] Ravindra N , Dillon LW , Gui G , et al. Persistent IDH mutations are not associated with increased relapse or death in patients with IDH‐mutated acute myeloid leukemia undergoing allogeneic hematopoietic cell transplant with post‐transplant cyclophosphamide. Bone Marrow Transplant. 2024;59(3):428‐430. doi:10.1038/s41409-023-02189-9 38182672

[215] Dinardo C , Marchione D , Heuser M , et al. Molecular measurable residual disease in patients with newly diagnosed m IDH1 acute myeloid leukemia treated with Ivosidenib + Azacitidine. Blood. 2023;142:4305. doi:10.1182/blood-2023-180549

[216] Montesinos P , Fathi AT , de Botton S , et al. Differentiation syndrome associated with treatment with IDH2 inhibitor enasidenib: pooled analysis from clinical trials. Blood Adv. 2024;8(10):2509‐2519. doi:10.1182/bloodadvances.2023011914 38507688 PMC11131052

[217] Fathi AT , DiNardo CD , Kline I , et al. Differentiation syndrome associated with Enasidenib, a selective inhibitor of mutant isocitrate dehydrogenase 2: analysis of a phase 1/2 study. JAMA Oncol. 2018;4(8):1106‐1110. doi:10.1001/jamaoncol.2017.4695 29346478 PMC5885269

[218] Norsworthy KJ , Mulkey F , Scott EC , et al. Differentiation syndrome with Ivosidenib and Enasidenib treatment in patients with relapsed or refractory IDH‐mutated AML: a U.S. Food and Drug Administration systematic analysis. Clin Cancer Res. 2020;26(16):4280‐4288. doi:10.1158/1078-0432.CCR-20-0834 32393603 PMC7442588

[219] Montesinos P , Bergua JM , Vellenga E , et al. Differentiation syndrome in patients with acute promyelocytic leukemia treated with all‐trans retinoic acid and anthracycline chemotherapy: characteristics, outcome, and prognostic factors. Blood. 2009;113(4):775‐783. doi:10.1182/blood-2008-07-168617 18945964

[220] Fruchtman H , Avigan ZM , Waksal JA , Brennan N , Mascarenhas JO . Management of isocitrate dehydrogenase 1/2 mutated acute myeloid leukemia. Leukemia. 2024;38(5):927‐935. doi:10.1038/s41375-024-02246-2 38600315 PMC11073971

[221] Wang W , Xue Y , Zhou S , Kuo A , Cairns BR , Crabtree GR . Diversity and specialization of mammalian SWI/SNF complexes. Genes Dev. 1996;10(17):2117‐2130. doi:10.1101/gad.10.17.2117 8804307

[222] Mashtalir N , D'Avino AR , Michel BC , et al. Modular organization and assembly of SWI/SNF family chromatin remodeling complexes. Cell. 2018;175(5):1272‐1288. doi:10.1016/j.cell.2018.09.032 30343899 PMC6791824

[223] Mashtalir N , Suzuki H , Farrell DP , et al. A structural model of the endogenous human BAF complex informs disease mechanisms. Cell. 2020;183(3):802‐817.e24. doi:10.1016/j.cell.2020.09.051 33053319 PMC7717177

[224] Kwon H , Imbalzano AN , Khavari PA , Kingston RE , Green MR . Nucleosome disruption and enhancement of activator binding by a human SW1/SNF complex. Nature. 1994;370(6489):477‐481. doi:10.1038/370477a0 8047169

[225] Zhang X , Li B , Li W , et al. Transcriptional repression by the BRG1‐SWI/SNF complex affects the pluripotency of human embryonic stem cells. Stem Cell Rep. 2014;3(3):460‐474. doi:10.1016/j.stemcr.2014.07.004 PMC4266000 25241744

[226] Nakayama RT , Pulice JL , Valencia AM , et al. SMARCB1 is required for widespread BAF complex‐mediated activation of enhancers and bivalent promoters. Nat Genet. 2017;49(11):1613‐1623. doi:10.1038/ng.3958 28945250 PMC5803080

[227] Raab JR , Resnick S , Magnuson T . Genome‐wide transcriptional regulation mediated by biochemically distinct SWI/SNF complexes. PLoS Genet. 2015;11(12):e1005748. doi:10.1371/journal.pgen.1005748 26716708 PMC4699898

[228] Alver BH , Kim KH , Lu P , et al. The SWI/SNF chromatin remodelling complex is required for maintenance of lineage specific enhancers. Nat Commun. 2017;8(1):14648. doi:10.1038/ncomms14648 28262751 PMC5343482

[229] Smith JJ , Xiao Y , Parsan N , et al. The SWI/SNF chromatin remodeling assemblies BAF and PBAF differentially regulate cell cycle exit and cellular invasion in vivo. PLoS Genet. 2022;18(1):e1009981. doi:10.1371/journal.pgen.1009981 34982771 PMC8759636

[230] Brownlee PM , Meisenberg C , Downs JA . The SWI/SNF chromatin remodelling complex: its role in maintaining genome stability and preventing tumourigenesis. DNA Repair. 2015;32:127‐133. doi:10.1016/j.dnarep.2015.04.023 25981841

[231] Kadoch C , Hargreaves DC , Hodges C , et al. Proteomic and bioinformatic analysis of mammalian SWI/SNF complexes identifies extensive roles in human malignancy. Nat Genet. 2013;45(6):592‐601. doi:10.1038/ng.2628 23644491 PMC3667980

[232] Mittal P , Roberts CWM . The SWI/SNF complex in cancer: biology, biomarkers and therapy. Nat Rev Clin Oncol. 2020;17(7):435‐448. doi:10.1038/s41571-020-0357-3 32303701 PMC8723792

[233] Reddy D , Bhattacharya S , Workman JL . mis. Cancer Metastasis Rev. 2023;42(2):455‐470. doi:10.1007/s10555-023-10102-5 37093326 PMC10349013

[234] Fiskus W , Piel J , Collins M , et al. BRG1/BRM inhibitor targets AML stem cells and exerts superior preclinical efficacy combined with BET or menin inhibitor. Blood. 2024;143(20):2059‐2072. doi:10.1182/blood.2023022832 38437498 PMC11830987

[235] Rago F , Rodrigues LU , Bonney M , et al. Exquisite sensitivity to dual BRG1/BRM ATPase inhibitors reveals broad SWI/SNF dependencies in acute myeloid leukemia. Mol Cancer Res. 2022;20(3):361‐372. doi:10.1158/1541-7786.MCR-21-0390 34799403

[236] Shi J , Whyte WA , Zepeda‐Mendoza CJ , et al. Role of SWI/SNF in acute leukemia maintenance and enhancer‐mediated Myc regulation. Genes Dev. 2013;27(24):2648‐2662. doi:10.1101/gad.232710.113 24285714 PMC3877755

[237] Kim H , Tan TK , Lee DZY , et al. Oncogenic dependency on SWI/SNF chromatin remodeling factors in T‐cell acute lymphoblastic leukemia. Leukemia. 2024;38(9):1906‐1917. doi:10.1038/s41375-024-02331-6 38969731

[238] DiNardo C . Preliminary Results from a Phase 1 Dose Escalation Study of FHD‐286, a Novel BRG1/BRM (SMARCA4/SMARCA2) Inhibitor, Administered As an Oral Monotherapy in Patients with Advanced Hematologic Malignancies. In: ASH. 2023 https://ash.confex.com/ash/2023/webprogram/Paper178090.html

[239] Oike T , Ogiwara H , Tominaga Y , et al. A synthetic lethality‐based strategy to treat cancers harboring a genetic deficiency in the chromatin remodeling factor BRG1. Cancer Res. 2013;73(17):5508‐5518. doi:10.1158/0008-5472.CAN-12-4593 23872584

[240] Sasaki M , Ogiwara H . Synthetic lethal therapy based on targeting the vulnerability of SWI/SNF chromatin remodeling complex‐deficient cancers. Cancer Sci. 2020;111(3):774‐782. doi:10.1111/cas.14311 31955490 PMC7060479

[241] Vangamudi B , Paul TA , Shah PK , et al. The SMARCA2/4 ATPase domain surpasses the bromodomain as a drug target in SWI/SNF‐mutant cancers: insights from cDNA rescue and PFI‐3 inhibitor studies. Cancer Res. 2015;75(18):3865‐3878. doi:10.1158/0008-5472.CAN-14-3798 26139243 PMC4755107

[242] Lee ECY , Reichl KD , Gopalsamy A . Synthetic lethality: targeting the SMARCA2 bromodomain for degradation in SMARCA4‐deficient tumors: a review of patent literature from 2019‐June 2023. Expert Opin Ther Pat. 2024;34(4):211‐229. doi:10.1080/13543776.2024.2355258 38742308

[243] Guo R , Dowlati A , Dagogo‐Jack I , et al. 603O first clinical results from a phase I trial of PRT3789: a first‐in‐class intravenous SMARCA2 degrader, in patients with advanced solid tumors with a SMARCA4 mutation. Ann Oncol. 2024;35:S483‐S484. doi:10.1016/j.annonc.2024.08.670

[244] Dermawan JK , Singer S , Tap WD , et al. The genetic landscape of SMARCB1 alterations in SMARCB1‐deficient spectrum of mesenchymal neoplasms. Mod Pathol. 2022;35(12):1900‐1909. doi:10.1038/s41379-022-01148-x 36088476 PMC9712236

[245] McBride MJ , Pulice JL , Beird HC , et al. The SS18‐SSX fusion oncoprotein hijacks BAF complex targeting and function to drive synovial sarcoma. Cancer Cell. 2018;33(6):1128‐1141. doi:10.1016/j.ccell.2018.05.002 29861296 PMC6791822

[246] Kadoch C , Crabtree GR . Reversible disruption of mSWI/SNF (BAF) complexes by the SS18‐SSX oncogenic fusion in synovial sarcoma. Cell. 2013;153(1):71‐85. doi:10.1016/j.cell.2013.02.036 23540691 PMC3655887

[247] Liu NQ , Paassen I , Custers L , et al. SMARCB1 loss activates patient‐specific distal oncogenic enhancers in malignant rhabdoid tumors. Nat Commun. 2023;14(1):7762. doi:10.1038/s41467-023-43498-3 38040699 PMC10692191

[248] Wang X , Wang S , Troisi EC , et al. BRD9 defines a SWI/SNF sub‐complex and constitutes a specific vulnerability in malignant rhabdoid tumors. Nat Commun. 2019;10(1):1881. doi:10.1038/s41467-019-09891-7 31015438 PMC6479050

[249] Michel BC , D'Avino AR , Cassel SH , et al. A non‐canonical SWI/SNF complex is a synthetic lethal target in cancers driven by BAF complex perturbation. Nat Cell Biol. 2018;20(12):1410‐1420. doi:10.1038/s41556-018-0221-1 30397315 PMC6698386

[250] Martin LJ , Koegl M , Bader G , et al. Structure‐based design of an in vivo active selective BRD9 inhibitor. J Med Chem. 2016;59(10):4462‐4475. doi:10.1021/acs.jmedchem.5b01865 26914985 PMC4885110

[251] Mancarella C , Morrione A , Scotlandi K . PROTAC‐based protein degradation as a promising strategy for targeted therapy in sarcomas. Int J Mol Sci. 2023;24(22):16346. doi:10.3390/ijms242216346 38003535 PMC10671294

[252] Sabnis RW . Novel compounds for targeted degradation of BRD9 and their use for treating cancer. ACS Med Chem Lett. 2022;13(1):17‐18. doi:10.1021/acsmedchemlett.1c00658 35059116 PMC8762736

[253] Clark PGK , Dixon DJ , Brennan PE . Development of chemical probes for the bromodomains of BRD7 and BRD9. Drug Discov Today Technol. 2016;19:73‐80. doi:10.1016/j.ddtec.2016.05.002 27769361

[254] Colarusso E , Chini MG , Bifulco G , Lauro G , Giordano A . Identification and development of BRD9 chemical probes. Pharm Basel. 2024;17(3):392. doi:10.3390/ph17030392 PMC10976250 38543178

[255] Shain AH , Pollack JR . The spectrum of SWI/SNF mutations, ubiquitous in human cancers. PLoS One. 2013;8(1):e55119. doi:10.1371/journal.pone.0055119 23355908 PMC3552954

[256] Xue Y , Meehan B , Fu Z , et al. SMARCA4 loss is synthetic lethal with CDK4/6 inhibition in non‐small cell lung cancer. Nat Commun. 2019;10(1):557. doi:10.1038/s41467-019-08380-1 30718506 PMC6362083

[257] Moison C , Chagraoui J , Caron MC , et al. Zinc finger protein E4F1 cooperates with PARP‐1 and BRG1 to promote DNA double‐strand break repair. Proc Natl Acad Sci U S A. 2021;118(11):e2019408118. doi:10.1073/pnas.2019408118 33692124 PMC7980444

[258] Yu L , Wu D . SMARCA2 and SMARCA4 participate in DNA damage repair. Front BiosciLandmark. 2024;29(7):262. doi:10.31083/j.fbl2907262 39082357

[259] Brodeur MN , Dopeso H , Zhu Y , et al. Interferon response and epigenetic modulation by *SMARCA4* mutations drive ovarian tumor immunogenicity. Sci Adv. 2024;10(49):eadk4851. doi:10.1101/2023.08.08.552544 39630912 PMC11616711

[260] Shen J , Ju Z , Zhao W , et al. ARID1A deficiency promotes mutability and potentiates therapeutic antitumor immunity unleashed by immune checkpoint blockade. Nat Med. 2018;24(5):556‐562. doi:10.1038/s41591-018-0012-z 29736026 PMC6076433

[261] Su P , Liu Y , Chen T , et al. In vivo CRISPR screens identify a dual function of MEN1 in regulating tumor‐microenvironment interactions. Nat Genet. 2024;56(9):1890‐1902. doi:10.1038/s41588-024-01874-9 39227744 PMC11387198

[262] Issa GC , Aldoss I , Thirman MJ , et al. Menin inhibition with Revumenib for. J Clin Oncol. 2025;43(1):JCO2400826. doi:10.1200/JCO.24.00826 PMC11687943 39121437

[263] Filippakopoulos P , Picaud S , Mangos M , et al. Histone recognition and large‐scale structural analysis of the human bromodomain family. Cell. 2012;149(1):214‐231. doi:10.1016/j.cell.2012.02.013 22464331 PMC3326523

[264] Filippakopoulos P , Knapp S . Targeting bromodomains: epigenetic readers of lysine acetylation. Nat Rev Drug Discov. 2014;13(5):337‐356. doi:10.1038/nrd4286 24751816

[265] Filippakopoulos P , Qi J , Picaud S , et al. Selective inhibition of BET bromodomains. Nature. 2010;468(7327):1067‐1073. doi:10.1038/nature09504 20871596 PMC3010259

[266] Dey A , Chitsaz F , Abbasi A , Misteli T , Ozato K . The double bromodomain protein Brd4 binds to acetylated chromatin during interphase and mitosis. Proc Natl Acad Sci U S A. 2003;100(15):8758‐8763. doi:10.1073/pnas.1433065100 12840145 PMC166386

[267] Jang MK , Mochizuki K , Zhou M , Jeong HS , Brady JN , Ozato K . The bromodomain protein Brd4 is a positive regulatory component of P‐TEFb and stimulates RNA polymerase II‐dependent transcription. Mol Cell. 2005;19(4):523‐534. doi:10.1016/j.molcel.2005.06.027 16109376

[268] Trojer P . Targeting BET bromodomains in cancer. Annu Rev Cancer Biol. 2022;6(1):313‐336. doi:10.1146/annurev-cancerbio-070120-103531

[269] Krönke J , Fink EC , Hollenbach PW , et al. Lenalidomide induces ubiquitination and degradation of CK1α in del(5q) MDS. Nature. 2015;523(7559):183‐188. doi:10.1038/nature14610 26131937 PMC4853910

[270] Krönke J , Udeshi ND , Narla A , et al. Lenalidomide causes selective degradation of IKZF1 and IKZF3 in multiple myeloma cells. Science. 2014;343(6168):301‐305. doi:10.1126/science.1244851 24292625 PMC4077049

[271] Wang B , Cao S , Zheng N . Emerging strategies for prospective discovery of molecular glue degraders. Curr Opin Struct Biol. 2024;86:102811. doi:10.1016/j.sbi.2024.102811 38598983

[272] Tan X , Huang Z , Pei H , Jia Z , Zheng J . Molecular glue‐mediated targeted protein degradation: a novel strategy in small‐molecule drug development. iScience. 2024;27(9):110712. doi:10.1016/j.isci.2024.110712 39297173 PMC11409024

[273] Li YD , Ma MW , Hassan MM , et al. Template‐assisted covalent modification underlies activity of covalent molecular glues. Nat Chem Biol. 2024;20(12):1640‐1649. doi:10.1038/s41589-024-01668-4 39075252 PMC11582070

[274] Bourgeois W , Cutler JA , Aubrey BJ , et al. Mezigdomide is effective alone and in combination with menin inhibition in preclinical models of KMT2A‐r and NPM1c AML. Blood. 2024;143(15):1513‐1527. doi:10.1182/blood.2023021105 38096371 PMC11033588

[275] Hansen JD , Correa M , Nagy MA , et al. Discovery of CRBN E3 ligase modulator CC‐92480 for the treatment of relapsed and refractory multiple myeloma. J Med Chem. 2020;63(13):6648‐6676. doi:10.1021/acs.jmedchem.9b01928 32130004

[276] Ross DM , Stevenson WS , Shortt J , Cooney J , Lewis I , Rienhoff HY Jr . Phase 1/2a trial of Bomedemstat with or without all‐ trans retinoic acid (ATRA) in advanced myeloid malignancies. Blood. 2023;142(1):6494. doi:10.1182/blood-2023-180924

[277] Foghorn Therapeutics Announces Clinical Data from Phase 1 Study of FHD‐286 in Metastatic Uveal Melanoma|Foghorn Therapeutics. 2024 https://ir.foghorntx.com/news-releases/news-release-details/foghorn-therapeutics-announces-clinical-data-phase-1-study-fhd/

[278] Agulnik M , Tap WD , Cote GM , et al. Initial results from a phase 1 study of CFT8634, a novel bifunctional degradation activating compound (or BIDAC™ degrader) of BRD9, in synovial sarcoma and SMARCB1‐null tumors.

[279] C4 Therapeutics, Inc . A phase 1–2 open‐label, multicenter study to characterize the safety and tolerability of CFT8634 in subjects with locally advanced or metastatic SMARCB1‐perturbed cancers, including synovial sarcoma and SMARCB1‐null tumors. 2024. https://clinicaltrials.gov/study/NCT05355753

[280] George TJ , Lee JH , Hosein PJ , et al. Results of a phase II trial of the PARP inhibitor, niraparib, in BAP1 and other DNA damage response pathway deficient neoplasms. J Clin Oncol. 2022;40(16_suppl):3122. doi:10.1200/JCO.2022.40.16_suppl.3122 PMC11616782 39626160

[281] Kim KB , Desprez P , Caressi C , et al. 1087P phase II study of niraparib in patients with advanced melanoma with homologous recombination pathway gene mutations. Ann Oncol. 2024;35:S719‐S720. doi:10.1016/j.annonc.2024.08.1155 40373259

[282] Eder JP . A Phase II Study of the PARP Inhibitor Olaparib (AZD2281) Alone and in Combination With AZD1775, AZD5363, or AZD6738 in Advanced Solid Tumors. 2022 https://clinicaltrials.gov/study/NCT02576444

[283] Banerjee S , Leary A , Stewart JR , et al. 34O ATR inhibitor alone (ceralasertib) or in combination with olaparib in gynaecological cancers with ARID1A loss or no loss: results from the ENGOT/GYN1/NCRI ATARI trial. ESMO Open. 2023;8(1):100814. doi:10.1016/j.esmoop.2023.100814

[284] Cote G . A Phase II Study of M6620 (VX‐970) in Selected Solid Tumors. 2023 https://clinicaltrials.gov/study/NCT03718091

[285] Swisher EM , Duska LR , Hamilton EP , et al. Abstract CT160: phase 1b/2a clinical trial of the oral BET inhibitor PLX2853 as monotherapy for ARID1A mutated gynecologic cancers and in combination with carboplatin for platinum resistant ovarian cance. Cancer Res. 2023;83(8_Suppl):CT160. doi:10.1158/1538-7445.AM2023-CT160

[286] O'Cearbhaill RE , Miller A , Soslow RA , et al. A phase 2 study of dasatinib in recurrent clear cell carcinoma of the ovary, fallopian tube, peritoneum or endometrium: NRG oncology/gynecologic oncology group study 0283. Gynecol Oncol. 2023;176:16‐24. doi:10.1016/j.ygyno.2023.06.021 37418832 PMC10529107

[287] Chi SN , Yi JS , Williams PM , et al. Tazemetostat for tumors harboring SMARCB1/SMARCA4 or EZH2 alterations: results from NCI‐COG pediatric MATCH APEC1621C. J Natl Cancer Inst. 2023;115(11):1355‐1363. doi:10.1093/jnci/djad085 37228094 PMC11009504

[288] Chi SN , Bourdeaut F , Casanova M , et al. Update on phase 1 study of tazemetostat, an enhancer of zeste homolog 2 inhibitor, in pediatric patients with relapsed or refractory integrase interactor 1‐negative tumors. J Clin Oncol. 2022;40(16_suppl):10040. doi:10.1200/JCO.2022.40.16_suppl.10040

[289] Gounder M , Schöffski P , Jones RL , et al. Tazemetostat in advanced epithelioid sarcoma with loss of INI1/SMARCB1: an international, open‐label, phase 2 basket study. Lancet Oncol. 2020;21(11):1423‐1432. doi:10.1016/S1470-2045(20)30451-4 33035459

[290] Upadhyaya SA , Campagne O , Billups CA , et al. Phase II study of alisertib as a single agent for treating recurrent or progressive atypical teratoid/rhabdoid tumor. Neuro‐Oncol. 2023;25(2):386‐397. doi:10.1093/neuonc/noac151 35652336 PMC9925713

